# Identification and functional validation of HPV-mediated hypermethylation in head and neck squamous cell carcinoma

**DOI:** 10.1186/gm419

**Published:** 2013-02-05

**Authors:** Matthias Lechner, Tim Fenton, James West, Gareth Wilson, Andrew Feber, Stephen Henderson, Christina Thirlwell, Harpreet K Dibra, Amrita Jay, Lee Butcher, Ankur R Chakravarthy, Fiona Gratrix, Nirali Patel, Francis Vaz, Paul O'Flynn, Nicholas Kalavrezos, Andrew E Teschendorff, Chris Boshoff, Stephan Beck

**Affiliations:** 1 UCL Cancer Institute, University College London, 72 Huntley Street, London, WC1E 6DD; 2 Head and Neck Centre, University College London Hospitals NHS Trust, Euston Road, London, NW1 2PG; 3 Department of Histopathology, University College London Hospitals NHS Trust, Rockefeller Building, University Street, London, WC1E 6JJ

## Abstract

**Background:**

Human papillomavirus-positive (HPV+) head and neck squamous cell carcinoma (HNSCC) represents a distinct clinical and epidemiological condition compared with HPV-negative (HPV-) HNSCC. To test the possible involvement of epigenetic modulation by HPV in HNSCC, we conducted a genome-wide DNA-methylation analysis.

**Methods:**

Using laser-capture microdissection of 42 formalin-fixed paraffin wax-embedded (FFPE) HNSCCs, we generated DNA-methylation profiles of 18 HPV+ and 14 HPV- samples, using Infinium 450 k BeadArray technology. Methylation data were validated in two sets of independent HPV+/HPV- HNSCC samples (fresh-frozen samples and cell lines) using two independent methods (Infinium 450 k and whole-genome methylated DNA immunoprecipitation sequencing (MeDIP-seq)). For the functional analysis, an HPV- HNSCC cell line was transduced with lentiviral constructs containing the two HPV oncogenes (*E6 *and *E7*), and effects on methylation were assayed using the Infinium 450 k technology.

**Results and discussion:**

Unsupervised clustering over the methylation variable positions (MVPs) with greatest variation showed that samples segregated in accordance with HPV status, but also that HPV+ tumors are heterogeneous. MVPs were significantly enriched at transcriptional start sites, leading to the identification of a candidate CpG island methylator phenotype in a sub-group of the HPV+ tumors. Supervised analysis identified a strong preponderance (87%) of MVPs towards hypermethylation in HPV+ HNSCC. Meta-analysis of our HNSCC and publicly available methylation data in cervical and lung cancers confirmed the observed DNA-methylation signature to be HPV-specific and tissue-independent. Grouping of MVPs into functionally more significant differentially methylated regions identified 43 hypermethylated promoter DMRs, including for three cadherins of the Polycomb group target genes. Integration with independent expression data showed strong negative correlation, especially for the cadherin gene-family members. Combinatorial ectopic expression of the two HPV oncogenes (*E6 *and *E7*) in an HPV- HNSCC cell line partially phenocopied the hypermethylation signature seen in HPV+ HNSCC tumors, and established *E6 *as the main viral effector gene.

**Conclusions:**

Our data establish that archival FFPE tissue is very suitable for this type of methylome analysis, and suggest that HPV modulates the HNSCC epigenome through hypermethylation of Polycomb repressive complex 2 target genes such as cadherins, which are implicated in tumor progression and metastasis.

## Background

Head and neck cancer is the sixth most common cancer worldwide, with an incidence of around 600,000 cases per year, with rising trends particularly in young people [1,2]. Despite recent advances in the treatment and in the understanding of its biology, the 5-year survival rate of 50% for patients with head and neck cancer has on the whole remained largely unchanged for the past three decades, with only some advances since the 1990s [3]. The most common type of head and neck cancer is squamous cell carcinoma (HNSCC). Human papillomavirus (HPV) represents a major independent risk factor for HNSCC. HPV is particularly associated with oropharyngeal carcinoma, of which 20 to 50% test positive for the HPV-16 subtype, with expression of the *E6 *and *E7 *viral oncogenes [4-6]. HPV-positive (HPV+) HNSCC represents a distinct molecular, epidemiologic, and clinical condition [7,8], and responds better than HPV-negative (HPV-) to chemotherapy and radiotherapy (82% response rate for HPV+ versus 55% for HPV- cases) and has a better disease-free and overall survival (95% versus 62% at 2 years) [9]. Individuals with HPV+ HNSCC have a lower rate of second primary tumors, and a decreased cumulative incidence of relapse [10,11]. Thus, knowledge of a patient's HPV status offers the possibility of stratifying such patients for treatment and of elucidating the mechanisms underlying the virus-associated advantage in drug response and survival in HNSCC.

The causes responsible for the different clinical behavior between HPV+ and HPV- tumors remain poorly understood. Numerous studies comparing gene expression patterns of HPV+ and HPV- cancers have shown different profiles for the two groups [12-16]. It is therefore likely that virus-mediated changes in both the genome and epigenome account for this differing clinical behavior. Deep exome sequencing of HPV+ and HPV- HNSCC recently confirmed mutations in *TP53 *as a potential genomic stratifier for HPV status [17,18]. Analysis of the epigenome is more complex, and the majority of studies have therefore focused on the methylome, because DNA methylation is the most accessible epigenetic modification in clinical samples [19].

Changes in DNA methylation play a key role in malignant transformation, leading to the silencing of tumor-suppressor genes and overexpression of oncogenes [20]. In virus-induced cancers, methylation changes have been described in both the host [21,22] and viral [23,24] methylomes. A recent study [25] comparing two HPV+ with two HPV- HNSCC cell lines showed that HPV infection is associated with changes in methylation of host genes, and led us to embark on a comprehensive study of HPV-mediated DNA methylation in HNSCC tumors. The study identified five Polycomb repressive complex 2 (PRC2) targets among the hypermethylated promoters. Polycomb group (PcG) proteins are transcriptional repressors, which modify histone tails to reversibly suppress genes required for differentiation. These proteins play a major role in neoplasia [26], and their oncogenic function is associated with a well-established role in stem-cell maintenance. Stem-cell PcG targets were shown to be 12 times more likely than non-targets to have cancer-specific promoter hypermethylation [27-29], supporting the theory of a stem-cell origin of cancer [30].

Based on these findings, we hypothesized that HPV modulates the epigenome in HNSCC, and set out to test this by comprehensive methylome analysis of HPV+ and HPV- primary tumors and cell lines (with the HPV- tissues serving as the control group, similar to a variety of previous expression studies in the field of HNSCC [14,15].. In addition, we aimed to phenocopy any HPV-mediated DNA-methylation signature by ectopic expression of HPV oncogenes in HPV- HNSCC cell lines.

## Methods

### Ethics approval

Ethics approval for this study was granted by the ethics committee of University College London/University College London Hospitals (UCL/UCLH; (reference number 04/Q0505/59), and informed consent was obtained where required.

### Patient samples and clinical data

We obtained 107 archival formalin-fixed paraffin wax-embedded tissue (FFPE) oropharyngeal cancer samples from the Department of Histopathology (UCLH) and tested for HPV status. Of these, 21 HPV+ and 21 HPV- age-matched samples were selected for methylation analysis (see Additional File 1, Figure S1 for workflow of FFPE sample preparation and selection). Histological diagnosis was confirmed by an experienced histopathologist, and correlated with clinical findings (see Additional File 2, Table S1). Furthermore, three fresh-frozen (FF) HPV+ and HPV- HNSCC samples (see Additional File 2, Table S2) were obtained from the UCLH Head and Neck Tumour Bank.

### Assessment of HPV status

HPV status was determined by CDKN2A (p16) immunostaining of the corresponding FFPE blocks, and confirmed by *E6 *quantitative (q)PCR on DNA extracted from both FF and FFPE samples. This combination of tests has been shown to have 97% sensitivity and 94% specificity, and to be the best discriminator of favorable outcome (see Additional File 2, Table S3) [31].

#### p16 staining

p16 staining was performed using a fully automated immunohistochemistry staining system (Bond™-III; Leica Microsystems, Inc., Buffalo Grove, IL, USA). Sections 3 μm thick were cut from a total of 82 FFPE blocks set from HNSCC samples (see Additional File 1, Figure S1), and were prepared for p16 staining. Using the staining system, slides were dewaxed (Bond Dewax Solution; Leica Microsystems) in accordance with the manufacturer's recommendation (protocol '*D'). Antigen retrieval was conducted using the accompanying solution (Bond ER1; Leica Microsystems) for 30 minutes in accordance with the manufacturer's protocol (*H1(30)). Staining was then performed using the accompanying detection kit (Bond Polymer Refine Kit; Leica Microsystems) in accordance with the manufacturer's protocol (15,8,8), using the pre-diluted p16 antibody clone (E6H4™; Roche mtm Laboratories, Heidelberg, Germany), and a negative reagent control (CINtec; Ventana Medical Systems, Inc., Tucson, AZ, USA). The stained slides were examined by two experienced histopathologists, and scored as described previously [32],. Subsequently, p16 positive areas of HPV-positive tumor samples were subjected to laser-capture microdissection (so the relative amount of p16-positive tumor cells used for testing should be close to 100%) and tumor samples showing a mixed staining pattern (*n *= 8) were excluded from further analysis (see Additional File 1, Figure S1).

#### E6 qPCR

DNA from both laser-capture microdissected HPV+ and HPV- HNSCC samples was used for E6 qPCR (see Additional File 1, Figure S1). E6 qPCR was optimized using primers and TaqMan probes (and using glyceraldehyde-3-phosphate dehydrogenase (GAPDH) as a housekeeping control), to test for the DNA regions of interest.

Primer and probe sequences have been published previously ([33]; see Additional File 2, Table S4; see Additional File 2, Table S5). DNA was amplified using qPCR with 25 μl 2× Buffer A (ABgene, Epsom, Surrey, UK), 0.3 μmol/l forward primer, 0.3 μmol/l reverse primer, and 0.15 μmol/l TaqMan probe, in a total volume of 50 μl. As controls, a housekeeping gene (GAPDH) and water sample were included in each PCR setup. qPCR was performed using a PCR cycler (Realplex Mastercycler; Eppendorf, Stevenage, UK) applying the following qPCR program: denaturation at 95°C for 15 minutes, followed by 40 cycles of 95°C for 15 seconds and 60°C for 60 seconds, with no extension step. All reactions were run in duplicate (two reactions at 1× concentration and two reactions with 1:10 dilution). The E6 qPCR is specific for HPV type 16, which is found in the vast majority of HPV-positive HNSCC specimens. A selected number of our samples were also tested for HPV type 18 and for low-risk HPV types (including HPV type 6 and HPV type 11, causative agents in laryngeal papillomatosis) by *in situ *hybridization (analysis was performed by UCL Advanced Diagnostics, University College London, London, UK). The results on all tested samples were negative, and HPV type 16 was the only HPV type that was detected.

### Laser-capture microdissection

Laser-capture microdissection (LCM) was carried out on slides (PALM MembraneSlide 1.0 PEN; Zeiss Microimaging, Munich, Germany) using an automated processor (PALM Microbeam™ system; Zeiss Microimaging). Depending on tumor size and pathology annotation, clusters of tumor cells were microdissected from one or more slides of the same FFPE block. For the HPV+ and HPV- samples, only the respective p16-positive and p16-negative tumor areas were dissected. The captured cells were estimated to contain 80% or more tumor cells.

### DNA extraction

DNA was extracted using commercial kits from the FF tumor samples (QIAamp DNA Blood Mini Kit (Qiagen GmBH, Hilden, Germany) and the laser-dissected FFPE samples (QIAamp DNA FFPE Tissue Kit; Qiagen GmBH).

### Genome-wide methylation analysis

DNAs were prepared in a total volume of 20 μl (1 μg of FF and cell-line DNAs and 2 μg of FFPE DNA per sample) using a previously optimized protocol [34], in conjunction with two commercial kits (REPLIg FFPE kit; catalog number 150243; Qiagen GmBH) and EZ DNA Methylation kit (catalog number D5001; Zymo Research Corp, Orange, CA, USA)). The latter kit was modified to improve bisulfite conversion efficiency by inclusion of a cyclic denaturation step as described previously [34]. A microarray platform (Infinium HumanMethylation450 BeadChips; Illumina Inc., San Diego, CA, USA) was used, which was processed by the UCL Genomics Core Facility in accordance with the manufacturer's recommendation. The scanned data and image output files were managed with Genomestudio software (version 1.9.0; Illumina).

R statistical software (version 2.14.0 [35]) was used for the subsequent data analysis. Raw data were subjected to a stringent quality-control analysis as follows. Samples showing reduced coverage were removed, and only probes with detection levels above background across all samples were kept (detection *P *< 0.01), resulting in a raw data matrix of 439,385 probes and 32 samples (18 HPV+ and 14 HPV-). This raw data matrix was then subjected to a principal component analysis to determine the nature of the largest components of variation. We used random matrix theory (RMT) to estimate the number of significant components of variation [36,37].

The 450 k BeadChips contain two types of probes (type 1 and 2) which have slightly different profiles. Although there have been attempts to normalize for that difference [38], we found that both the proposed normalization methods and the in-house methods that we developed overcorrected the data, leading to worse performance as evaluated using a rigorous training-test set partition strategy. Thus, in our supervised analysis, we treated both types of design probes equally, and carried out *a posteriori *testing for a potential skew favoring type 1 probes. Although there were only 1,075 type 1 probes among the top 2757, this amounted to an over-enrichment, with an odds ratio of 1.48 (*P *< 1 × 10^16^). However, after correcting for differences in CpG density between type 1 and type 2 probes, the enrichment odds ratio favoring type 1 probes was significantly reduced to 1.13 (*P *= 0.03 approximately). Thus, there was no substantial skew favoring type 1 probes, and we found that normalizing for the design using the peak-based correction method of Dedeurwader only led to overcorrection and increased technical variability (see Additional File 1, Figure S2).

All normalized and raw 450 k methylation data were submitted to the Gene Expression Omnibus (GEO; National Center for Biotechnology Information, Bethesda, MD. USA) in accordance with the instructions provided (GEO accession numbers: GSE38266, GSE38268, GSE38270 and GSE38271).

### Hypermethylation signature

To quantify the strength of the association and to adjust for multiple testing, we estimated the false-discovery rate (FDR) using the q-value procedure [39]. Because the analytical q-value estimates assume independence of the underlying tests, which does not necessarily apply to neighboring probes that are spatially correlated, we also estimated the FDR using a permutation approach that preserves the potential correlation structure of proximal probes. However, empirical and analytical FDR estimates were in close agreement (see Additional File 1, Figure S3). Both procedures estimated approximately 2,750 methylation variable positions (MVPs) at FDR of less than 0.01, that is, less than 1% of the 2,757 probes are expected to be false positives. Probes from the X and Y chromosomes were removed when obtaining the methylation signature.

### Copy number variation analysis

Copy number variation (CNV) analysis was performed on the DNA of the three HPV+ and three HPV- FF HNSCC samples using a genotyping array (HumanOmni1-Quad BeadChip; Illumina). This analysis was required for the normalization of the methylated DNA immunoprecipitation sequencing (MeDIP-seq) data. CNV data were analyzed using Genome Studio software (Illumina).

### Whole-genome methylation analysis with MeDIP-seq

DNAs from three HPV+ and three HPV- fresh-frozen HNSCC samples were subjected to autoMeDIP-seq as previously described [40], using a master mix (NEBNext DNA Sample Prep Mastermix; New England Biolabs, Beverly, MA, USA) for the library preparation and magnetic beads (MagMeDIP; Diagenode, Liege, Belgium) for the immunoprecipitation. Adequate enrichment of the methylated DNA fraction (compared with input) was quality controlled using qPCR. Following adapter-mediated PCR, the library was subjected to size selection (300 to 350 bp) using low melting-point agarose gels. The excised fraction was quality controlled by qPCR. Cluster generation and 36 bp end sequencing was performed, by the UCL Genomics Core Facility, using an genome analyzer (GAIIX; Illumina) in accordance with the manufacturer's recommendation.

The data were analyzed using the MeDUSA pipeline [41]. Reads were aligned to the reference genome (Human assembly GRCh37) using the alignment software BWA (version 0.5.8) [42], with default parameters. Filtering was performed using SAMtools (version 0.1.9) [43] to remove erroneously mapped and low-quality (score of < 10) reads. Only reads forming a correctly aligned pair were kept. A final filtering step removed potential PCR artifacts by discarding all but one read-pair within groups of non-unique fragments (see Additional File 2, Table S6). Read quality was ascertained using FastQC [44] and the Bioconductor package MEDIPS (version 1.0.0) [45]. Probes from the 450 k BeadChips located within CpG island regions were isolated, and these sites were extended to create 500 bp windows. Absolute methylation scores for each of these regions were calculated from our MeDIP read files using MEDIPS. Methylation scores were calculated for each extended probe site using default values.

All normalized and raw MeDIP-seq data were submitted to GEO (NCBI) in accordance with the instructions provided (GEO accession numbers: GSE38263).

### Integration of obtained methylation data with publicly available methylation data on cervical cancer and lung cancer

R statistical software v2.15.1 [35] was used for pre-processing of data and for classic MDS)(principal coordinates analysis). MDS was used to visualize HPV+ and HPV- HNSCC methylation signatures within methylation datasets obtained from an HPV-induced cancer type (cervical cancer; GSE32861) and an smoking-induced cancer type (lung cancer; GSE30759). In detail, for the lung-cancer dataset, 27,578 probe IDs for 59 lung-cancer samples (from a total of 118: 59 lung-cancer and 59 adjacent-tissue samples) were selected. For the cervical-cancer dataset, 27,578 probe IDs for 48 cervical-cancer samples (from 63 samples in total) were selected. The relevant methylation data from the processed dataset of 18 HPV+ and 14 HPV- HNSCCs were extracted (439,385 probe IDs for 32 HNSCC samples). Probe IDs culminated in data matrices of 27,300 probe IDs (59 lung-cancer samples), 26,871 probe IDs (48 cervical-cancer samples), 439,385 probe IDs (18 HPV+ HNSCC samples), and 439,385 probe IDs (14 HPV- HNSCC samples). All the datasets were restricted to the common probe IDs (24,145 probe IDs) by the 'intersect' function in R. As outlined above, 2,757 MVPs were identified (using a Bayesian regularized t-statistics model) with an FDR of less than 0.01 in HPV+ HNSCC, compared with HPV- HNSCC. The respective probe IDs were intersected with the common probe IDs identified in each dataset (lung cancer, cervical cancer, HPV+ HNSCC, HPV- HNSCC). This resulted in 90 common probe IDs identified across all the datasets, and represents the selection of HPV-associated versus smoking-associated features tested. To check whether the same pattern was present in cervical cancer versus lung cancer, multidimensional scaling (MDS) of the samples was created using a Euclidean distance measure after scaling all common probe ID features, with the R 'cmdscale' function. A Wilcoxon rank sum test was used to test inter-sample distances between the evaluated datasets (the set of distances between HPV+ HNSCC and cervical-cancer samples was compared with the set of distances between HPV+ HNSCC and lung-cancer samples).

### Cell culture and production of SCC003 clones expressing HPV-16 oncogenes

HNSCC cell lines UPCI:SCC090 (HPV+), UPCI:SCC003 (HPV-), UPCI:SCC036 (HPV-) and PCI-30 (HPV-) (generous gifts from Dr Susanne Gollin and Dr Theresa Whiteside, University of Pittsburgh Cancer Institute, Pittsburgh, PA, USA). 93VU-147T (HPV+) (generous gift from Dr. Hans Joenje, VU Medical Center, Netherlands) and UM:SCC047 (HPV+) (generous gift from Dr Thomas Carey, University of Michigan, Ann Arbor, MI, USA) were used. All cell lines (see Additional File 2, Table S7) were maintained in DMEM supplemented with 10% FBS and penicillin/streptomycin. Clones of SCC003, expressing either empty vector control, HPV-16 *E7*, HPV-16 *E6*, or both HPV-16 *E6 *and *E7*, respectively, were generated by infection of the SCC003 cell line with retroviruses followed by single-cell cloning as follows. Viruses were produced by transfecting human embryonic kidney (HEK)293T cells with pLXSN (empty vector or containing the HPV-16 *E6, E7*, or *E6*&*E7 *cDNAs, kind gifts from Dr David Beach), together with pHIT- VSVG and MLV-gag/pol (kind gifts from Dr Juan Martin-Serrano) using polyethylenimine (Polysciences, Inc., Warrington, PA, USA), then 72 hours post-transfection, viruses were harvested by removal of the medium and filtration through 0.45 μm surfactant-free filters (Nalgene Nunc International Corporation, Rochester, NY, USA). The filtered virus stocks were either frozen at -80C or diluted 1:2 in DMEM with 10% FBS and 8 μg/ml hexadimethrine bromide (Polybrene; Sigma-Aldrich, St Louis, MO, USA to give a final concentration of 4 μg/ml, then added to SCC003 cells grown to a confluence of 40 to 50%. Following overnight incubation, the cells were washed to remove virus, and the medium was replaced with DMEM plus 10% FBS. At 48 to 72 hours post-infection, cells were passaged at a ratio of 1:5 into selection medium containing 400 μg/ml G418. Following death of all mock-infected cells (approx. 1 to 2 weeks), cells were removed from selection, and plated at limiting dilution in 96-well plates to generate single-cell clones. HPV-16 *E6 *and *E7 *qPCR was conducted as described previously [33,46]. To assess *E6 *and *E7 *expression levels in *E6-*transduced, *E7-*transduced, and *E6+E7-*transduced SCC003 cell-line clones (and empty vector controls), qPCR was performed on cDNA following reverse transcription (Superscript II; Invitrogen Corp., Carlsbad, CA, USA) of total RNA purified from cells (miRNeasy kit; Qiagen GmBH), in accordance with the manufacturers' recommendations.

### RNA extraction

For this experiment, three biological replicates of six HNSCC cell lines (SCC47, SCC90, 93VU, PCI30, SCC003, and SCC036), as described above, were grown in T75 flasks in DMEM supplemented with 10% FCS and 1% penicillin/streptomycin, then RNA was extracted from (RNeasy Mini kit; Qiagen GmBH) in accordance with the manufacturer's recommended protocols, with DNAse digestion. cDNA was synthesized from 500 ng RNA (SuperScript II cDNA synthesis kit; Invitrogen) in accordance with the manufacturer's instructions.

Primers were designed for *CDH8, PCDH10, DNMT1, DNMT3a *and *DNMT3b *using a primer design tool (Integrated DNA Technologies, Coralville, IA, USA). Sequences are shown in Table 1.

**Table 1:** Primers used for quantitative PCR.

Primer	Direction	Sequence 5'→3'
*DNMT3a*	Forward	CTGGGAGGAAGCGCAAG
	Reverse	CCATTGGGTAATAGCTCTGAGG
*DNMT3b*	Forward	CCCATTCGAGTCCTGTCATTG
	Reverse	TTGATATTCCCCTCGTGCTTC
*DNMT1*	Forward	GAAGTGAAGCCCGTAGAGTG
	Reverse	GGTGCTTTTCCTTGTAATCCTG
*CDH8*	Forward	CTCTTCACCGACTTACCTACTTG
	Reverse	ATGTTGAACTGCCTCTCCAG
*PCDH10*	Forward	GACAGTGAACAGGGAGATAGTG
	Reverse	TCAGAAGGGACAAAAGAAGGC

Master mixes were made up with SYBR Green (Applied Biosystems, Foster City, CA, USA), 0.2 μmol/l primers and 1 μl cDNA in 10 μl reactions, and qPCR was carried out on a thermal cycler (Realplex^4 ^Mastercycler; Eppendorf AG, Hamburg, Germany) for 10 minutes at 95°C, followed by 40 cycles of 15 seconds at 95°C and 60 seconds at 60°C. Relative expression was defined in terms of fold change of expression between the cluster of three negative cell lines relative to three positive cell lines for *CDH8 *and PCDH10, and *vice versa *for *DNMT3a, DNMT3b, and DNMT1 *using the ^ΔΔ^Ct method on Ct values obtained from qPCR. *P *values were calculated using the two-tailed Student's *t*-test across Ct values for the six cell lines from three independent experiments.

## Results

### HPV+ tumors have a distinct DNA-methylation signature

**Figure 1:**
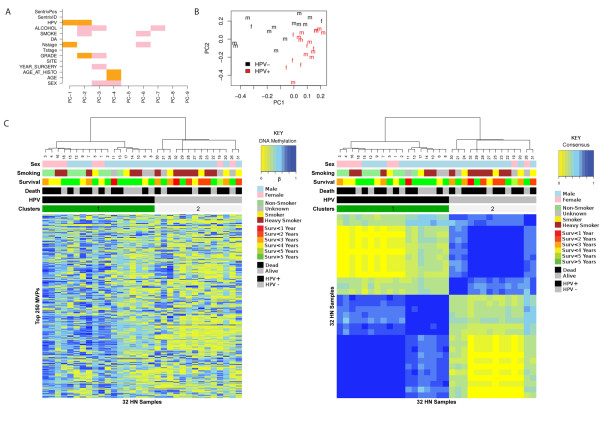
**Unsupervised analysis of the top 250 methylation variable positions (MVPs) in formalin-fixed, paraffin wax-embedded (FFPE) human papillomavirus-positive (HPV+) and HPV-negative (HPV-) tumor samples**. **(A) **Singular value decomposition: PC-k denotes the kth principal component, DA denotes survival at censoring date. The first two principal components PC-1 and PC-2 most strongly correlated with HPV status, whereas the remaining significant components associated with clinical parameters, including alcohol consumption, smoking, age, sex, tumor stage, and grade. No association was found with technical factors (such as Sentrix position and Sentrix ID) on the array. **(B) **The first two principal components clearly distinguish HPV status. HPV+ samples are plotted in black, HPV- samples are in red, and m/f indicates male/female. **(C) **Clusters inferred by the unsupervised consensus-clustering algorithm for the top 250 MVPs as found using the MAD estimator.

To investigate whether HPV+ and HPV- tumors have distinct epigenetic signatures, we performed genome-wide DNA-methylation profiling using the 450 k Illumina Infinium platform [47], which allows the methylation state of over 480,000 cytosine sites (mostly CpG sites) to be interrogated. FFPE samples from 21 HPV+ and 21 HPV- tumors were analyzed. Raw data were subjected to a stringent quality-control analysis (Methods). This resulted in a raw data matrix of 439,385 probes and 32 samples (18 HPV+ and 14 HPV-). This raw data matrix was then subjected to principal component analysis to determine the nature of the largest components of variation. Using RMT [36,37], we estimated a total of nine significant components of variation, which were mainly correlated with biological factors. The first two components correlated with HPV status, and confirming this, a scatter plot along these showed that samples segregated according to HPV status (Figure 1A, B). Importantly, there was no substantial variation associated with technical factors, including Sentrix position or identification (Figure 1A).

Because the first two principal components of these data corresponded most strongly with HPV status, we naturally expected that unsupervised clustering over the most variable probes would result in segregation of samples according to HPV status. As defined previously [48], such probes or CpG sites are referred to as MVPs and as hyper-MVPs or hypo-MVPs when directionality towards differential hypermethylation or hypomethylation has been ascertained. Segregation was confirmed by consensus clustering of the top 250 MVPs (Figure 1C).

**Figure 2:**
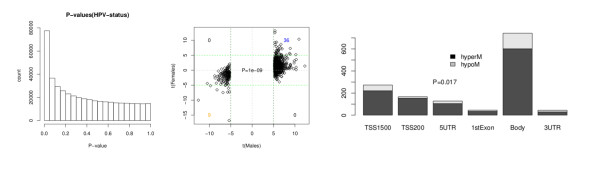
**Supervised differential methylation analysis reveals human papillomavirus-positive (HPV+) signature, showing a skew towards hypermethylation**. **(A) **Histogram of P-values from the supervised analysis a clear trend towards small significant *P *values. **(B) **Independence of HPV status and sex: scatter plot of t-statistics of individual CpGs reflecting HPV status (positive t-statistics indicate hypermethylation in HPV-infected samples). wiht *P *value computed using Wilcoxon rank sum test. **(C) **Methlyation status according to gene-feature annotation, showing a clear trend towards hypermethylation (*P *= 0.017). Gene features: TSS1500, TSS200, 5' untranslated region (UTR), first exon, gene body, 3' UTR.

Next, we performed a supervised analysis to ascertain the association between DNA methylation and HPV status. To rank probes, we used a Bayesian regularized *t*-statistics model [49], which has been used and validated in the context of DNA-methylation data [50]. Consistent with the previous unsupervised analysis, a histogram of *P*-values from the supervised analysis showed a clear trend towards small significant *P*-values (Figure 2A). Using two alternative procedures (*q*-values [39] and a permutation approach; see Methods), we found 2,757 MVPs with an FDR rate of less than 0.01, that is, less than 1% of the 2,757 MVPs are expected to be false positives. Of these 2,757 MVPs, the overwhelming majority (2,408; 87%) were hyper-MVPs in HPV+ samples, compared with HPV-, indicating that HPV infection is associated with widespread gain of DNA methylation. The MVPs indicating differential methylation between HPV+ and HPV- samples were independent of gender. Indeed, we derived ranked sets of MVPs associated with HPV status for the 24 men and 8 women separately, and the resulting statistics had high correlation (Figure 2B).

To investigate if the directional DNA-methylation changes were related to the position of the MVPs relative to the corresponding genes, we first categorized each MVP into one of six gene-feature groups (transcription start site (TSS)1500, TSS200, 5' untranslated region (UTR), first exon, body, and 3' UTR). We found that hyper-MVPs in HPV+ samples were preferentially located upstream or near the TSS or in gene bodies but not in first exons, whereas hypo-MVPs in HPV+ samples were preferentially located in gene bodies (Figure 2C). Taken together, these data clearly show that the HPV+ tumor samples have a distinct epigenetic signature, which shows a significant skew towards hypermethylation.

### HPV+ are heterogeneous, with a candidate CpG island methylator phenotype

**Figure 3:**
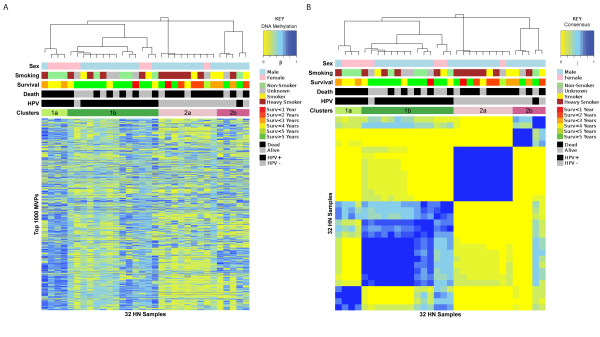
**Unsupervised analysis of the top 1,000 MVPs in formalin-fixed, paraffin wax-embedded (FFPE) human papillomavirus-positive (HPV+) and HPV-negative (HPV-) tumor samples**. **(A) **Consensus clustering identified four sub-groups in HPV+ and HPV- groups, revealing sub-group 1a as candidate CIMP within the HPV+ group. **(B) **Clusters inferred by the unsupervised consensus-clustering algorithm for the top 1,000 MVPs as found using the MAD estimator.

The enrichment of hyper-MVPs in HPV+ samples and the observation that many of these mapped to CpG islands suggested a possible association with CpG island methylator phenotype (CIMP) in these samples. To investigate this further, we performed consensus clustering over the top 1,000 MVPs, a procedure similar to the one used previously to discover CIMP phenotypes in breast and brain cancer [51,52]. The consensus clustering yielded four clusters, which still correlated with HPV status, but also showed heterogeneity within the HPV+ and HPV- subtypes (Figure 3). Specifically, we found two main sub-groups of HPV+ samples, with subtype 1a exhibiting higher methylation levels (Figure 3). This subtype was also characterized by higher average methylation levels when the MVPs were restricted to CpG islands (see Additional File 1, Figure S4), suggestive of CIMP. However, and in contrast to the CIMPs reported in breast, colon, and brain cancers, there was no evidence of a stronger correlated hypermethylation pattern in this subtype than in the rest of HPV+ tumors. Interestingly, the patient samples in our candidate CIMP cluster 1a all had poor outcome, exhibiting significantly shorter survival times compared with cluster 1b, which contained mostly samples from patients with good outcome (log-rank *P *= 0.001, Figure 3; see Additional File 1, Figure S4). It is noteworthy that there was no significant association with viral load, relative amount of p16-positive tumor cells, or expression of viral transcripts between these two sub-groups.

### Validation of the hypermethylation signature in HPV+ tumors

**Figure 4:**
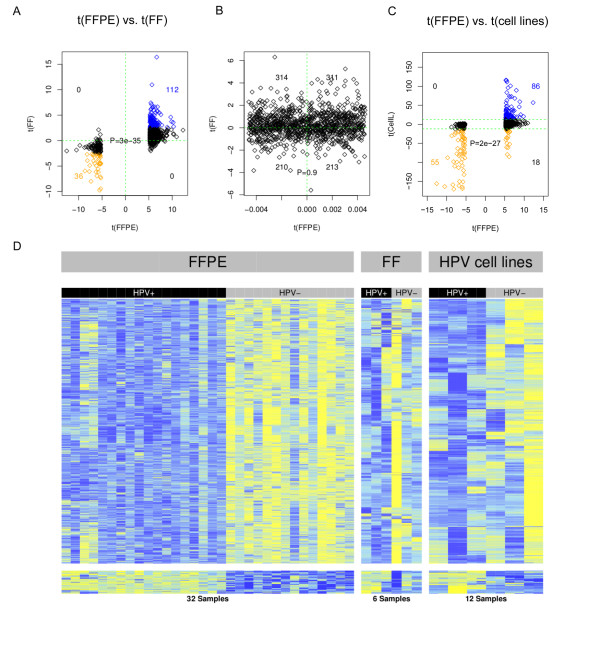
**Validation of human papillomavirus-positive (HPV+) and HPV-negative (HPV-) methylation signature**. **(A-C) **Validation of consistency of *t*-statistics between formalin-fixed, paraffin wax-embedded (FFPE) and **(A) **fresh-frozen (FF) samples, **(B) **FF control probes and **(C) **HPV+ against HPV- cell lines. **(D) **Heatmap representation of signature of consistent hypermethylated methylation variable positions (hyper-MVPs; top) and hypomethylated MVPs (hypo-MVPs; bottom) in the Infinium DNA-methylation data. The DNA-methylation (β) values are represented using a color scale from yellow (low DNA methylation) to blue (high DNA methylation) normalized across each MVP. The HPV+ head and neck squamous cell cancer (HNSCC) methylation signature contains 2,194 consistent hyper-MVPs and 74 consistent hypo-MVPs across all three datasets. The six HNSCC cell-line samples were run in duplicate.

To validate our findings, we performed 450 k Infinium profiling on six independent FF) samples (three HPV+ and three HPV-). All six samples passed our quality-control criteria (see Methods). We applied the same Bayesian supervised analysis to rank MVPs according to how well they discriminated the three HPV+ from the three HPV- samples. The overwhelming majority of MVPs that were significantly hypermethylated in HPV+ FFPE samples were also hypermethylated (and many significantly hypermethylated) in the HPV+ FF samples relative to HPV- samples. Comparing the regularized *t*-statistics obtained from the 32 FFPE samples with those obtained from the six FF samples, there was a very strong agreement (*P *= 3 × 10^-35^; Figure 4A). A control set of probes, which did not differ between HPV+ and HPV- FFPE samples, also did not correlate with HPV status in the FF set (Figure 4B). To further validate our findings and the 450 k technology, we compared the 450 k methylation values for CpG islands with the methylation scores calculated from MeDIP-seq using the MEDIPS package, testing the three HPV+ FF and three HPV- FF samples. We found strong agreement between the two methods (see Additional File 1, Figure S5).

Next, we investigated if the DNA-methylation changes associated with HPV status were also present in HPV- infected HNSCC cell lines. HPV *t*-statistics between the FFPE and HPV cell-line experiments correlated strongly (Fisher test, *P *= 2 × 10^-27^, Figure 4C). Changes in absolute mean beta value (Δβ) between HPV+ and HPV- cell lines were substantially larger than the changes detected in FFPE (paired Wilcoxon test *P *= 7 × 10^-15^), and again larger than in FF tumor samples (paired Wilcoxon test *P *= 3 × 10^-13 ^see Additional File 1, Figure S6A). In conclusion, we identified 2,194 consistent hyper-MVPs and 74 consistent hypo-MVPs across all three experiments (FFPE HNSCCs, FF HNSCCs, and HNSCC cell lines; Figure 4D). This confirms that our HPV+ hypermethylation signature obtained from FFPE samples was validated in an independent set of HNSCC samples, as well as in HPV+ HNSCC cell lines, and indicates a strong association of the observed methylation signature and HPV status. Consistent with the observed hypermethylation phenotype in HPV+ tumors and cell lines, real-time qPCR analysis showed increased mRNA expression of both the *de novo *DNA methyltransferase, DNMT3A (as described previously [25]) and the maintenance DNA methyltransferase DNMT1 in HPV+ cell lines, compared with HPV- cell lines (see Additional File 1, Figure S7).

### Ectopic expression of the HPV-16 oncogene *E6 *partially phenocopies the hypermethylation signature

To functionally validate our obtained HPV+ hypermethylation signature, we infected an HPV- HNSCC cell line with lentiviral vectors containing either or both of the HPV-16 oncogenes *E6 *and *E7*. After confirmation of ectopic expression of these HPV oncogenes in three clones of each cell line (see Additional File 2, Table S8), we performed DNA-methylation profiling on *E6*-infected, *E6*&*E7*-infected and *E7*-infected clones relative to empty vector controls.

**Figure 5:**
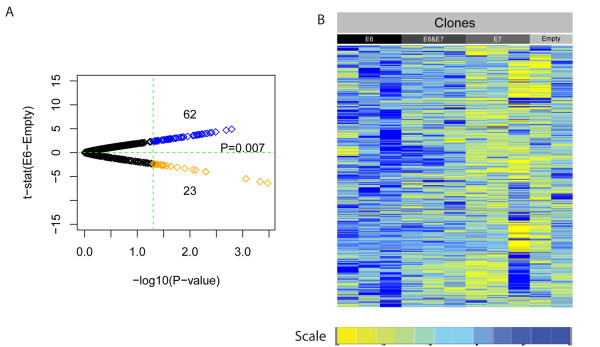
**Validation of consistent hypermethylated methylation variable positions (hyper-MVPs) in E6 and E6&E7 infected cell-line clones**. **(A) **The formalin-fixed, paraffin wax-embedded (FFPE) hyper-MVP signature consistent with E6 (infected with E6 or E6&E7) versus empty vector controls (Monte Carlo *P*= 0.007). Volcano plot shows t-statistics of E6 versus empty clones plotted against log_10 _FFPE *P *values. **(B) **Heat-map representation of consistent hyper-MVPs in clones infected with E6, E6&E7, or E7 and empty vector controls. Yellow indicates relative hypomethylation in HPV+ samples and blue indicates hypermethylation (MVPs normalized across samples).

The skew towards hypermethylation (seen in the described experiment on FFPE HNSCCs) was confirmed to be highly significant in *E6 *and *E6*&*E7 *clones against the background probability of hypermethylation (there was widespread hypermethylation in *E6 *and *E6*&*E7 *clones) (Figure 5A, Monte Carlo, *P *= 0.007). The distribution of methylation changes in *E6, E6*&*E7*, and *E7 *clones compared with controls is illustrated (see Additional File 1, Figure S8). In contrast to E6 and E6&E7, E7 was shown not to contribute to this hypermethylation signature (comparison of clones infected with *E7 *compared with controls; one-sided Wilcoxon, *P *= 1). The skew towards hypermethylation was significantly larger for *E6 *than for *E6*&*E7*, consistent with the lower expression levels of *E6 *in *E6*&*E7*-co-infected clones compared with *E6-*infected clones (see Additional File 2, Table S8). The results of the entire experiment are summarized in two graphs (Figure 5A, B). In conclusion, ectopic expression of E6 (but not E7) in HPV- HNSCC cell-line clones partially phenocopies the hypermethylation signature seen in HPV+ HNSCC tumors.

### Meta-analysis of HPV+ and HPV- HNSCC and of publicly available methylation data for cervical and lung cancer

**Figure 6:**
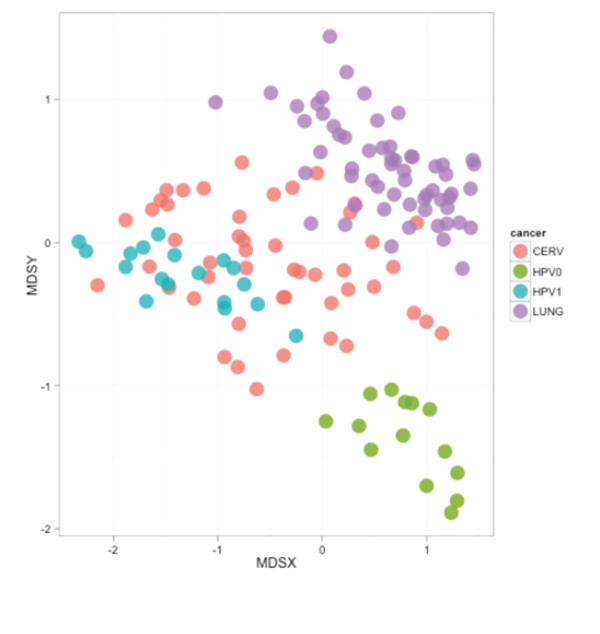
**Multidimensional scaling using the four datasets**. These datasets were comprised of 48 cervical-cancer samples (CERV; pink), 59 lung-cancer samples (LUNG; purple), 18 human papillomavirus-positive (HPV+) head and neck squamous cell cancer (HNSCC) samples (HPV1; light-blue) and 14 HPV-negative (HPV-) HNSCC samples (HPV0; green), using a selection of HPV-associated versus smoking-associated features identified by comparing HPV+ versus HPV- HNSCC.

To test further the effect of HPV on DNA methylation, methylation data obtained from the 18 HPV+ and 14 HPV- HNSCC samples were integrated with publicly available methylation data on HPV-induced versus smoking-induced cancer, 48 cervical-cancer samples [53] and 59 lung-cancer samples [54]. Using a selection of HPV-associated versus smoking-associated features (see Methods), identified by comparing HPV+ with HPV- HNSCC, MDS of the datasets using a simple Euclidean distance measure was applied, and distances were plotted. An overlap of cervical-cancer samples and HPV+ HNSCC samples was found (Figure 6). Significance of this observation was further tested using a Wilcoxon rank sum test on inter-sample distances. When focusing upon HPV+/HPV- methylation signatures, the methylation pattern of cervical-cancer samples was more closely related to the HPV+ signature seen in HNSCC (*P *< 2.2 × 10^-16^). This suggests that HPV induces a distinct methylation signature that is independent of tissue-specific DNA methylation.

### Enrichment of PRC2 targets, especially members of the cadherin superfamily, within the hypermethylation signature

To find consistent targets across all the data sets (FFPEs, FFs, and HPV+ cell lines), we assigned all of the consistent hyper-MVPs and hypo-MVPs identified above to genes, and ran a gene-set enrichment analysis. The hyper-MVPs, which make up the majority (96.7%) of MVPs, identified several SUZ12 and PRC2 targets (see Additional File 2, Table S9), including multiple members of the cadherin superfamily. Indeed, there was significant (11 probes in total, Fisher exact test, *P *= 4 × 10^-7^) enrichment of hyper-MVPs within the cadherin genes. This was also the case in the top 1000 MVPs (Fisher *P *= 0.0003). We tested the possible biological effects of these cadherins in three separate ways, by showing that 1) their methylation state was sufficient to accurately cluster samples in accordance with HPV status, 2) they have consistent and significant hypermethylation across their promoter regions (see section on differentially methylated regions (DMRs); and 3) this promoter hypermethylation associates with decreased gene expression in existing data (see Expression section). Using the *k*-medoids clustering algorithm (*pam *in the R package *survival*), we found that the 11 cadherin-annotated probes within the top 1,000 MVPs were sufficient to detect HPV status (84% correctly classified (27/32), Fisher exact test, *P *= 0.0002). These 11 MVPs mapped to CpG islands, shores, or shelves of six cadherin genes (*CDH8, CDH15, PCDH8, PCDH9, PCDH10*, and *PCDHB3)*. The remaining 3.3% hypo-MVPs were enriched for two gene sets previously shown to display upregulation of gene expression in HPV+ head and neck cancers [16,14]. Among the top 100 hits, we also found consistent enrichment of genes involved in DNA replication and binding, the mitogen-activated protein kinase pathway and E2F targets (see Additional File 2, Table S10).

To assess the MVP associations in a more biologically relevant context, we grouped them into DMRs if at least three (range three to seven) had correlated differential methylation levels within the TSS200 promoter region. TSS200 was chosen because it was most significantly positively (*P *= 2.4 × 10^-5^) enriched category of the six tested (see Additional File 1, Figure S9). Applying this filter, the 2,194 consistent hyper-MVPs mapped to 906 distinct genes, with 416 having at least three probes in their respective TSS200 regions. From these 416 genes, we derived 43 hypermethylated TSS200 DMRs (γβ > 0.1) across FFPE HNSCCs, FF HNSCCs, and HNSCC cell lines; all Wilcoxon paired *P *values < 0.05). A sample-permutation approach yielded an expected 4.4 false positives (see Methods). HPV cell lines showed the largest changes in mean TSS200 hypermethylation, significantly larger than FFPE (paired Wilcoxon, *P *= 5 × 10^-05^) which showed significant hypermethylation relative to FF (paired Wilcoxon, *P *= 1 × 10^-06^; see Additional File 1, Figure S6B). Using the same approach for the 74 consistent hypo-MVPs, we derived five hypomethylated TSS200 DMRs. Exemplar profiles of a hyper-DMR for *CDH8 *and a hypo-DMR for *MEI1 *(Figure 6_ highlight the increasing power to detect MVPs and DMRs dependent on cell-type purity (cell line > laser-capture microdissected FFPE > FF). All DMRs associated with cadherin genes had sample-permutation estimated *P *values of less than 0.05 ((or profiles of these, see Additional File 1, Figure S10). In summary, we found 43 genes with promoter hypermethylation consistently across all datasets (permutation FDR 10%) including multiple cadherin genes and other PRC2 targets. In addition, we found five genes (*SNTB1, CYP7B1, MEI1, ICA1, and FAM163A*) with hypomethylated promoter DMRs.

### Integration with publicly available gene-expression data

For additional functional evidence of the effect of DNA-methylation changes on gene expression, we compared our methylation differences between HPV+ and HPV- FFPE tumor samples with publicly available gene-expression data [14]. The top 500 MVPs mapping to CpG islands were compared with the differential expression *t*-statistics of their associated genes. We found a significant negative correlation (Fisher test, *P *= 2 × 10^-18^; Figure 8A). A list of genes with consistent TSS200 DMRs across all datasets (FFPE HNSCCs, FF HNSCCs, HNSCC cell lines), and which also exhibited differential gene expression in the independent Pyeon gene-expression dataset, is shown in Figure 8B. Among these were three cadherin genes (*CDH8, PCDH10 *and *PCDHB11*). These data are consistent with cadherin genes being targets for HPV-mediated hypermethylation and transcriptional silencing in HNSCC.

### CDH 8 and PCDH10 are hypermethylated and silenced in HPV+ HNSCC cell lines

To confirm that genes with promoters that are differentially methylated between HPV+ and HPV- cell lines are also differentially expressed, we carried out qPCR for *CDH8 *and *PCDH10*. We found that CDH8 and PCDH10 were significantly overexpressed in our panel of three HPV- HNSCC cell lines relative to three HPV+ cell lines, correlating with hypermethylation in the latter (see Additional File 1, Figure S11).

## Discussion

The findings reported here represent the most comprehensive epigenetic study of a virus-induced cancer to date, and the first to validate the existence of an HPV-mediated DNA-methylation signature in HPV+ HNSCC. Supported by extensive validation using independent samples and different methods, the signature showed a clear skew towards hypermethylation, which was most prominent at promoter regions (defined by TSS200). However, there was also significant hypomethylation at gene bodies, which, together with promoter hypermethylation, is a clear hallmark of gene silencing [55]. It is well documented, for instance, that hypermethylation of the promoter region of tumor-suppressor genes plays an important role in cellular transformation [20], and indeed, we found consistent hypermethylation (defined by both hyper-MVPs and hyper-DMRs) in the promoter regions of such genes, as well as in a candidate CIMP in the HPV+ samples. CIMPs have been reported for a number of cancers, including neuroblastoma [56], colon cancer [57], brain cancer [52], and breast cancer [51]. Except for neuroblastoma and possibly few other cancers, CIMP has been associated with a favorable clinical outcome. HPV+ HNSCC are also associated with a more favorable outcome [9] but the candidate CIMP found in the current study was only present in a sub-group of four HPV+ patients, who all had a shorter survival time and recorded death. To our knowledge, this is the first time that a CIMP (albeit a candidate CIMP) has been reported for HNSCC, representing a second example of CIMP being associated with potentially less favorable clinical outcome. Furthermore, we were able to show that our signature (defined by top 1,000 MVPs) was independent of gender and predictive for smoking status and length of survival, confirming previous findings [7,9,58].

**Figure 7:**
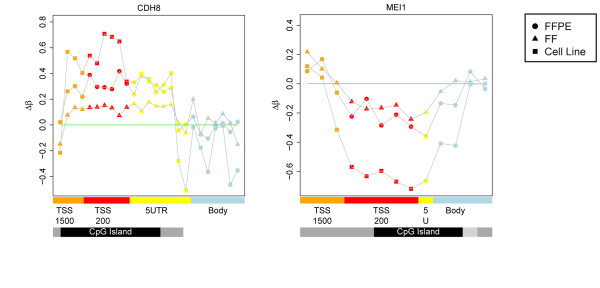
**Exemplar profiles of a hypermethylated differentially methylated region (hyper-DMR) for CDH8 and a hypomethylated DMR (hypo-DMR) for MEI1**. Comparison of DMR profiles obtained from formalin-fixed, paraffin wax-embedded (FFPE) head and neck squamous cell cancers (HNSCCs), fresh-frozen (FF) HNSCCs, and HNSCC cell lines. The profiles clearly show the increasing power to detect methylation variable positions and differentially methylated regions (DMRs) is dependent on cell-type purity (cell line > laser-capture microdissected FFPE > FF). Feature annotation is as provided by BeadChip, and methylation values are color-coded accordingly: TSS1500, orange (1500 bp to 200 bp upstream of the transcription start site (TSS)); TSS200, red (200 bp upstream of the TSS); 5' untranslated region (UTR), yellow; gene body, blue; CpG islands, black; CpG shores, grey; and CpG shelves, light grey.

The inclusion of multiple sample types (FFPE, FF, and cell lines) in the validation part revealed an important observation, with direct implications for projects with an epigenetic biomarker component, such as ICGC [59], IHEC [60], OncoTrack [61] and others. Although FF samples have emerged as the gold standard for the genomic analysis of cancer, our data show that archival FFPE samples may be superior for certain epigenomic analyses, particularly when combined with LCM, as illustrated in Figure 7. The largest differences in DNA-methylation levels were consistently found in cell lines, followed by laser-microdissected FFPE, followed by FF. This general trend was expected because DNA methylation is known to be cell type-specific but the evident high level of confounding cellular heterogeneity (resulting in dilution of the respective MVP/DMR signals) in carefully biobanked FF samples is nevertheless noteworthy.

**Figure 8:**
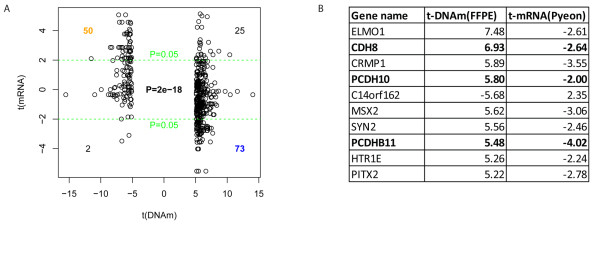
**Integration of DNA-methylation data with public gene-expression data**. **(A) **DNA methylation correlates with decreased gene expression: scatter plot of t-statistics between human papillomavirus-positive (HPV+) and HPV-negative (HPV-) formalin-fixed, paraffin wax-embedded (FFPE) cancer samples (top 500 differentially methylated MVPs restricted to CpG islands) shows significant anti-correlation between DNA methylation and gene expression. Gene expression-data were taken from Pyeon *et al*., [14]. **(B) **List of top 10 anti-correlated targets: Differentially methylated genes in promoter region (TSS200) which also exhibit differential gene expression in the independent Pyeon gene-expression dataset. 3 Cadherin genes were found among the top 10 hits (bold).

The most interesting finding arising from the gene set enrichment analysis is the enrichment of numerous members of the cadherin superfamily, which are targets of PRC2 and are implicated in many cancers and cancer-specific processes [62], including epithelial to mesenchymal transition (EMT), a process by which carcinomas become invasive and acquire the ability to metastasize [63]. Notable examples include *E-Cadherin *(*CDH1*), *T-Cadherin *(*CDH13*) and *Proto-Cadherin 10 *(*PCDH10*) which are recognized tumor-suppressor genes, and have been found to be hypermethylated in a number of human cancers [62]. Among the 49 PRC2 targets (defined by consistent hyper-MVPs) identified here were 10 genes of the cadherin superfamily in HPV+ HNSCC, including *CDH8 *and *CDH13 *(both also hypermethylated in cervical cancer [64], *CDH18, CDH19, CDH23, PCDH10, PCDH15, PCDHB1, PCDHB4, and PCDHB15*. Moreover, the 11 MVPs in 6 cadherin genes identified among the top 1,000 MVPs by unsupervised clustering analysis of FFPE HNSCC samples, warrant further investigation as potential biomarkers because they clustered our HPV+ and HPV- samples according to HPV status with high accuracy. Although we were able to show that DNA-methylation analysis is suitable for patient stratification according to HPV status, it was not more effective than mutation analysis or immunostaining with p16. Therefore, combinatorial testing may be the clinically most effective way to stratify patients for HPV in the future.

We obtained two lines of evidence with respect to functional support for the identified hypermethylation signature. First, we were able to partially phenocopy the signature by ectopic expression of the two HPV oncogenes *E6 *and *E7 *in an HPV- HNSCC cell line. Combinatorial analysis showed that *E6 *is the main viral effector gene. The underlying mechanism remains unknown, and is subject to future work such as analysis of cross-talk between *E6 *and DNA methyltransferases, effect of *E6 *on *TP53*, and number and distribution of viral integration sites into the host genome and the viral methylome itself. Second, we integrated publicly available expression data with our DNA-methylation data [14]. Among the top 10 anti-correlated (high promoter methylation and low expression) genes were three of the cadherins, namely *CDH8, PCDH10*, and *PCDHB11*. The inverse scenario (low promoter methylation and high expression) was also found, and both are likely to contribute to the different clinical behavior of HPV+ and HPV- HNSCC with regard to survival and response to therapy. Linking these two lines of evidence suggests a possible mechanism whereby HPV could drive tumor progression by promoting EMT [63] through epigenetic silencing of cadherins, in addition to its established role in tumor initiation.

## Conclusions

This work significantly advances our understanding of the epigenetic dynamics at genomic loci targeted by oncogenic viruses as shown here for loci associated with the infection of HPV in HNSCC. Based on the previously established finding that patients with HPV+ HNSCC have a better prognosis than do patients with HPV- HNSCC, it is tempting to speculate that this advantage may be partly epigenetically mediated. Our results certainly implicate DNA methylation in this process. If confirmed, targeted reprogramming of the identified HPV-mediated hypermethylation signature (or parts of it) in patients who suffer from HPV-cancer offers a potential translational application for our findings. Although still at an early experimental stage, targeted reprogramming has recently been reported, including in cancer cells [65,66]. In the longer term, these data will contribute to the identification of diagnostic and prognostic markers and of putative therapeutic targets.

## Abbreviations

CIMP: CpG island methylator phenotype; DMEM: Dulbecco's modified Eagle's medium; DMR: Differentially methylated region; DNMT: DNA methyltransferase; EMT: Epithelial to mesenchymal transition; FBS: Fetal bovine serum; FDR: False-discovery rate; FF: fresh-frozen; FFPE: Formalin-fixed, paraffin wax-embedded; GADPH: Glyceraldehyde-3-phosphate dehydrogenase; HEK: Hhuman embryonic kidney; HNSCC: Head and neck squamous cell cancer; HPV: Human papillomavirus; HPV+: HPV-positive; HPV-: HPV-negative; MDS: Multi-dimensonal scaling; MVP: Methylation variable position; PcG: Polycomb group proteins; PRC2: Polycomb repressive complex 2; qPCR: Quantitative polymerase chain reaction; RMT: Random matrix theory; TSS: transcription start site; UTR: untranslated region.

## Competing interests

The authors declare that they have no competing interests.

## Authors' contributions

All authors contributed to the interpretation of data and to the writing of the manuscript

ML, AET, CB, and SB were responsible for tudy design and conceptualization of the study; ML, AF, TF, CT, HKT, LB, NP, FG, FV, PO'F, and NK for sample preparation, tumor collection, and technical work; AJ for histology: AET, GW, ML, and JW for computational biology; and ML, AET, GW, and JW for figures and tables. All authors read and approved the final version.

## Supplementary Material

Additional file 1 **Supplemental figures**. Click here for file

Additional file 2 **Supplemental tables**. Click here for file
