# Supplementary material for: Identification and functional validation of HPV-mediated hypermethylation in head and neck squamous cell carcinoma
Source: Genome Med. 2013 Feb 5;5(2):15. doi: 10.1186/gm419 (PMC3706778; doi:10.1186/gm419)
Supplement: Additional file 1 — Supplemental figures. [file gm419-S1.DOCX]

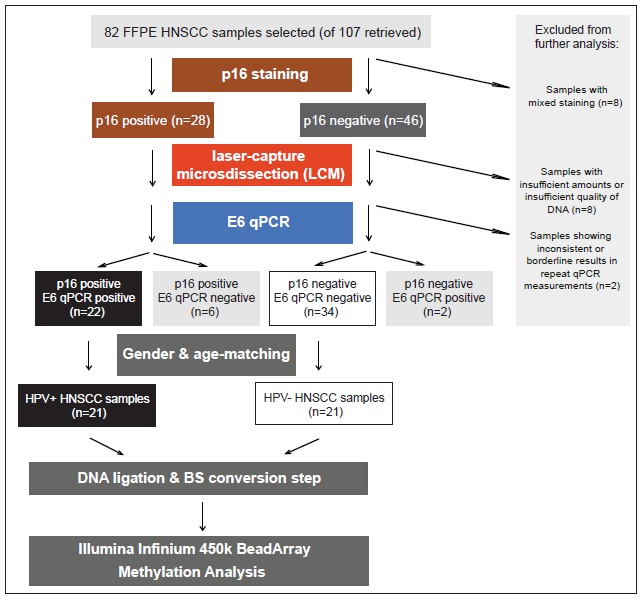


**Additional File 1, Figure S1:** **Workflow of FFPE sample preparation and selection.** After identification and retrieval of FFPE blocks, p16 staining and E6 qPCR was performed. 8 samples were excluded as they showed mixed p16 staining, further 8 samples were excluded as LCM yielded insufficient amounts or quality of DNA. 2 further samples were excluded, showing inconsistent or borderline results in repeat E6 qPCR measurements. Assuming E6 qPCR represents the gold standard, 22 samples were true positives and 34 samples were true negatives. 6 samples showed a false positive result and two showed a false negative result. Twenty-one HPV+ HNSCC samples (black) and 21 HPV- HNSCC samples (white) were then selected for the final analysis (Illumina Infinium 450k BeadArray Methylation Analysis), after having been selected according to gender and age-matching requirements, amount of DNA available and quality of DNA used.


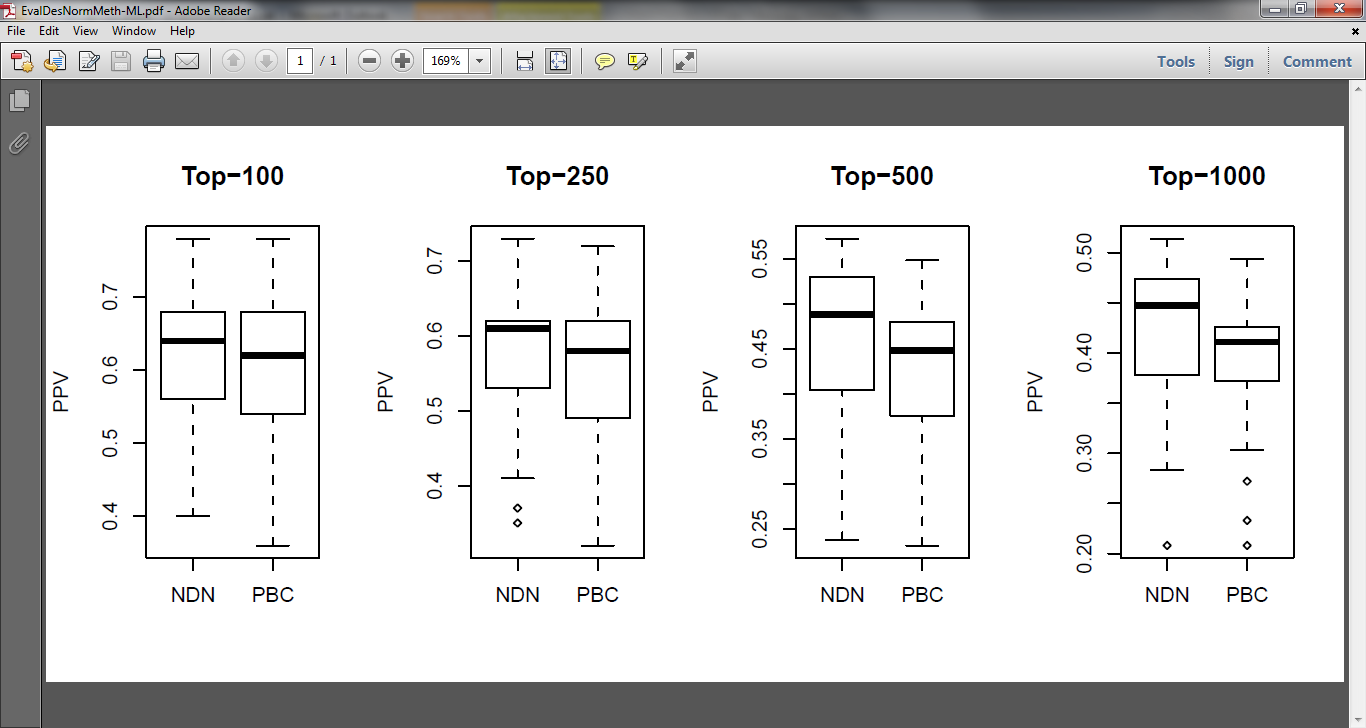


**Additional File 1, Figure S2: Peak-Based Correction (PBC) does not improve predictive power in FFPE HNSCC samples.** Positive predictive value (PPV, y-axis) for the case where no design normalisation is performed (NDN) against PBC normalisation. The PPV is estimated in a test set, where features have been ranked and selected from a mutually exclusive training set. The boxplots are over 25 distinct training/test set choices. This shows that PBC increases variability within phenotypes resulting in worse performance. The PPV is estimated as the fraction of the selected features that have a *P*-value < 0.05 in the test and preserving the same directionality as in training set.

**Additional File 1, Figure S3: Close agreement of analytic and permutation-based FDR estimates.** False discovery rate (FDR) of FFPE probes estimated by (1) red: analytic *q*-value procedure of Storey-Tibshirani and (2) black: using a permutation approach preserving the potential correlative structure of proximal probes (empirical). NP = number of probes, NFP = Number of False Positives.

A


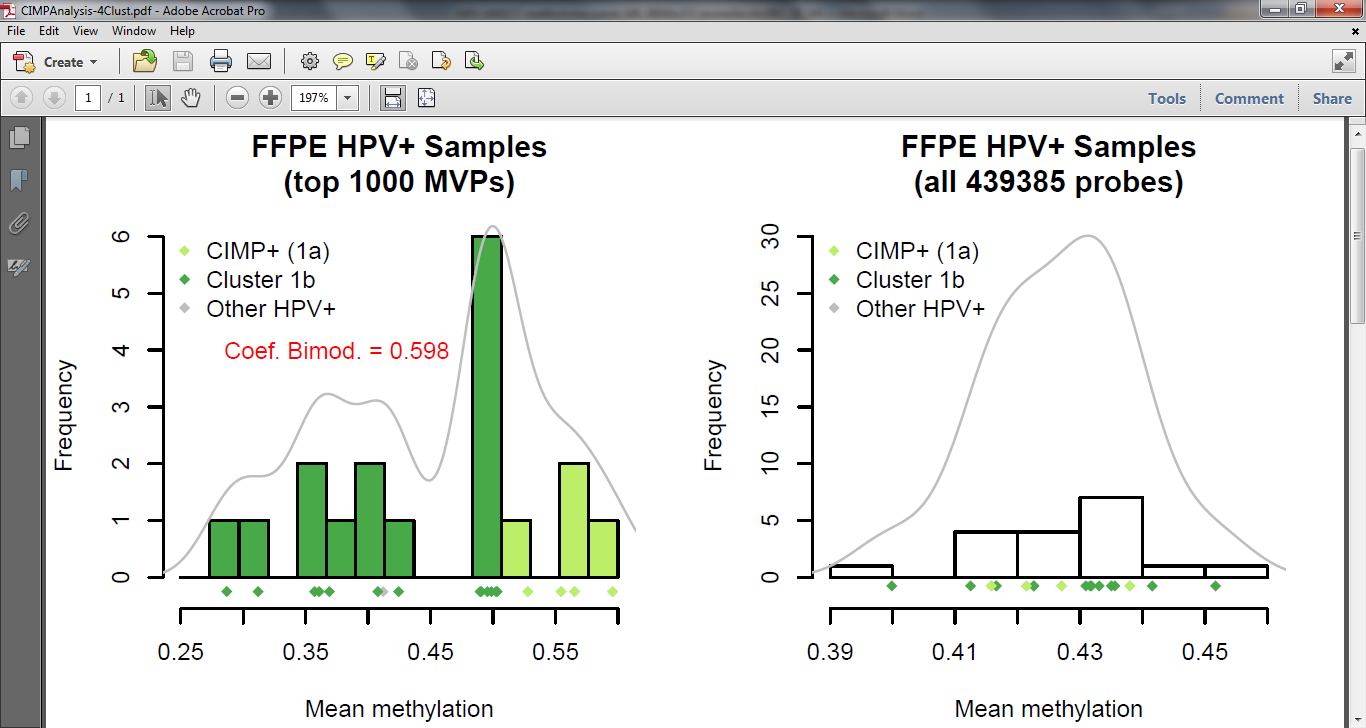


B


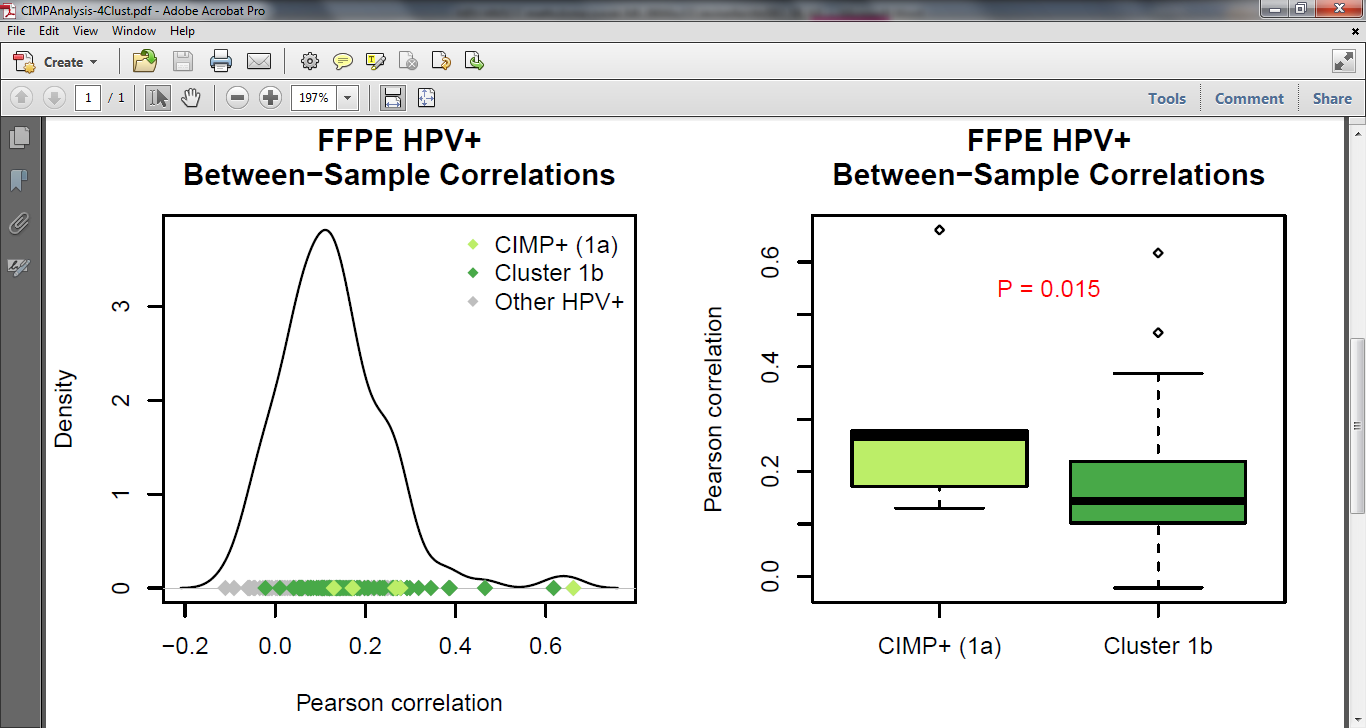


C


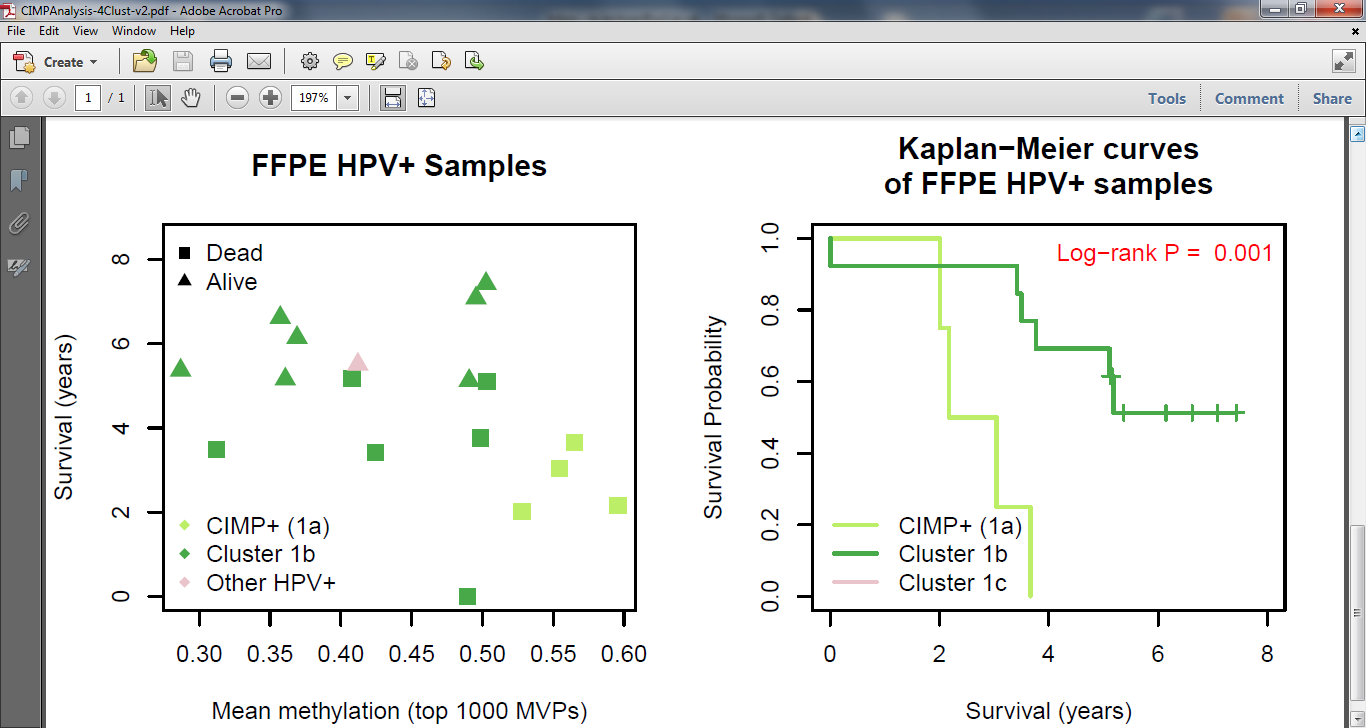


**Additional File 1, Figure S4: Testing for the presence of a HPV+ CIMP phenotype.**

A) Distribution of mean methlyation values across the top 1000 MVPs in HPV+. As illustrated, there was a slight bimodality (coefficient of bimodality = 0.6, for which 0.555 ~ uniform and 1 ~ Bernoulli) in the distribution of mean methlyation values across the top 1000 MVPs in HPV+, with significant hypermethylation of the CIMP+ cluster (1a, light green). Across all probes, no multimodality was observed, nor any significant separation of mean methylation levels between the CIMP+ cluster and the large HPV+ positive cluster with lower methylation levels (1b, dark green). All points of the distribution are displayed below the histograms (black) and density plots (grey). B) Significant separation (one sided *P* = 0.015) of between-sample correlation distributions of the CIMP+ cluster (1a) and cluster 1b. C) Association of mean methylation levels with survival independent of HPV status. There was an anticorrelation between mean levels across the top 1000 MVPs and survival time. Comparing the overall survival of patients in the CIMP+ cluster (1a) with patients in HPV+ cluster 1b, the HPV+ CIMP+ patients showed a significantly worse outcome (Log-rank *P* = 0.001).

**
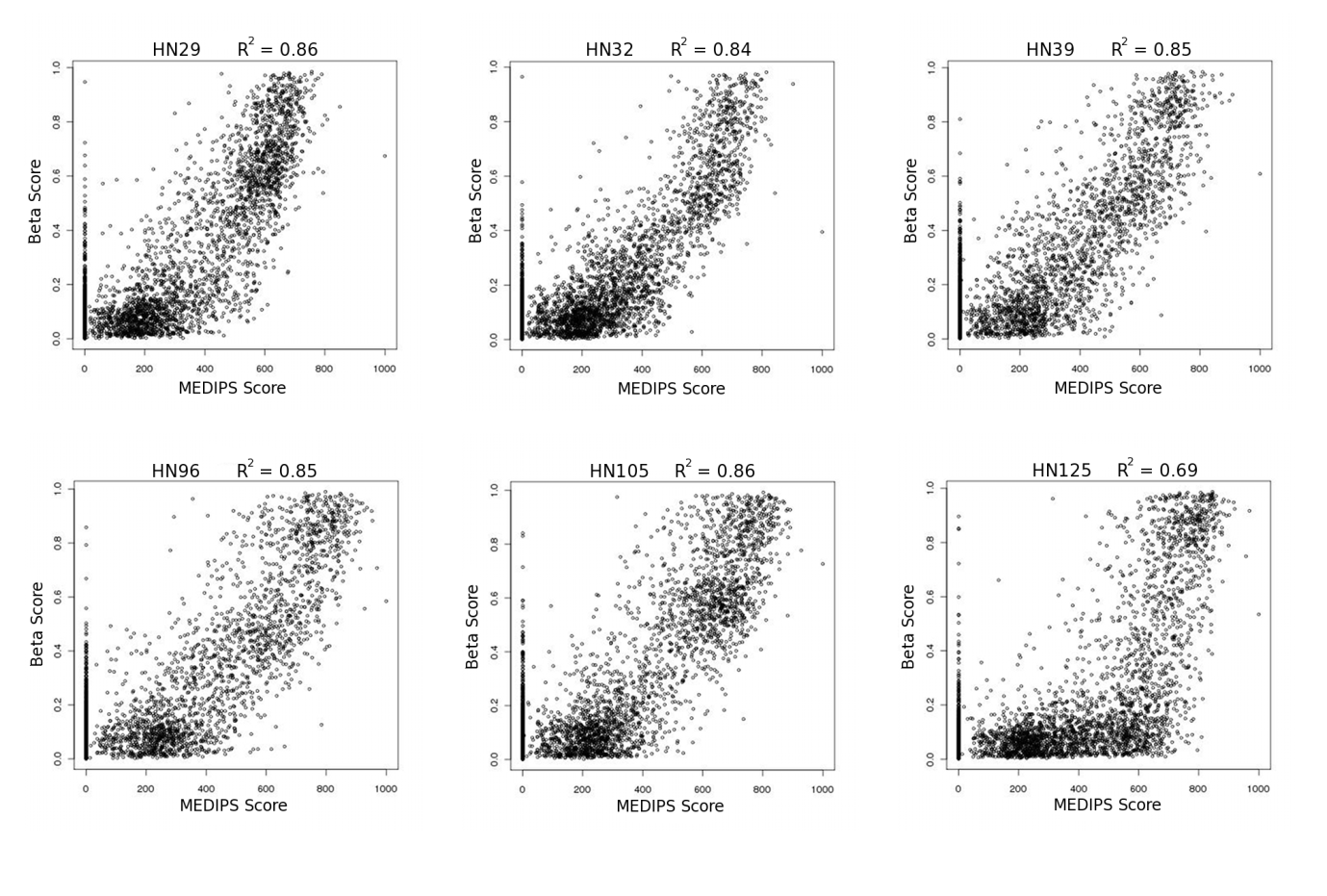
Additional File 1, Figure S5:** **Validation of the 450k technology by MeDIP-Seq.** Methylation score calculated from MeDIP-Seq using the MEDIPS package (MEDIPS Score) and beta values for CpG islands (Beta Score), testing the 3 HPV+ FF and 3 HPV– FF samples. Correlation coefficients (Pearson) illustrated above each scatterplot.

A


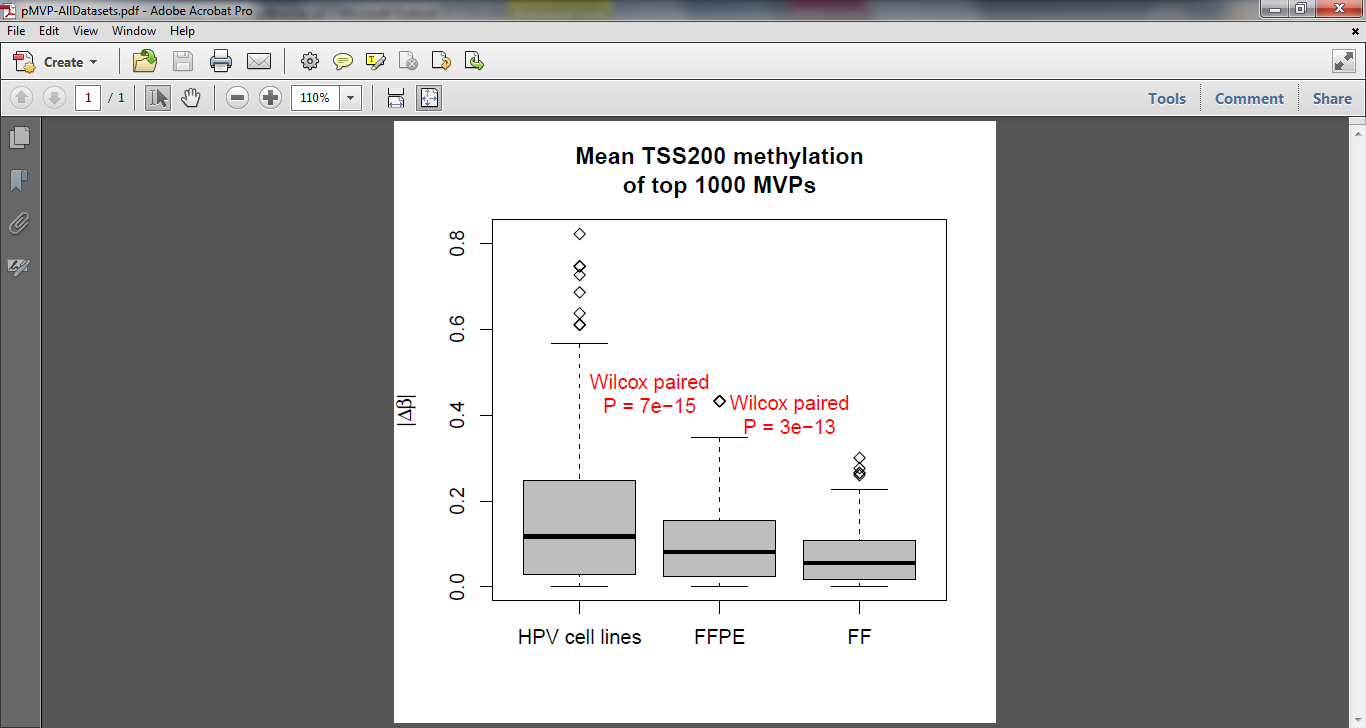


B


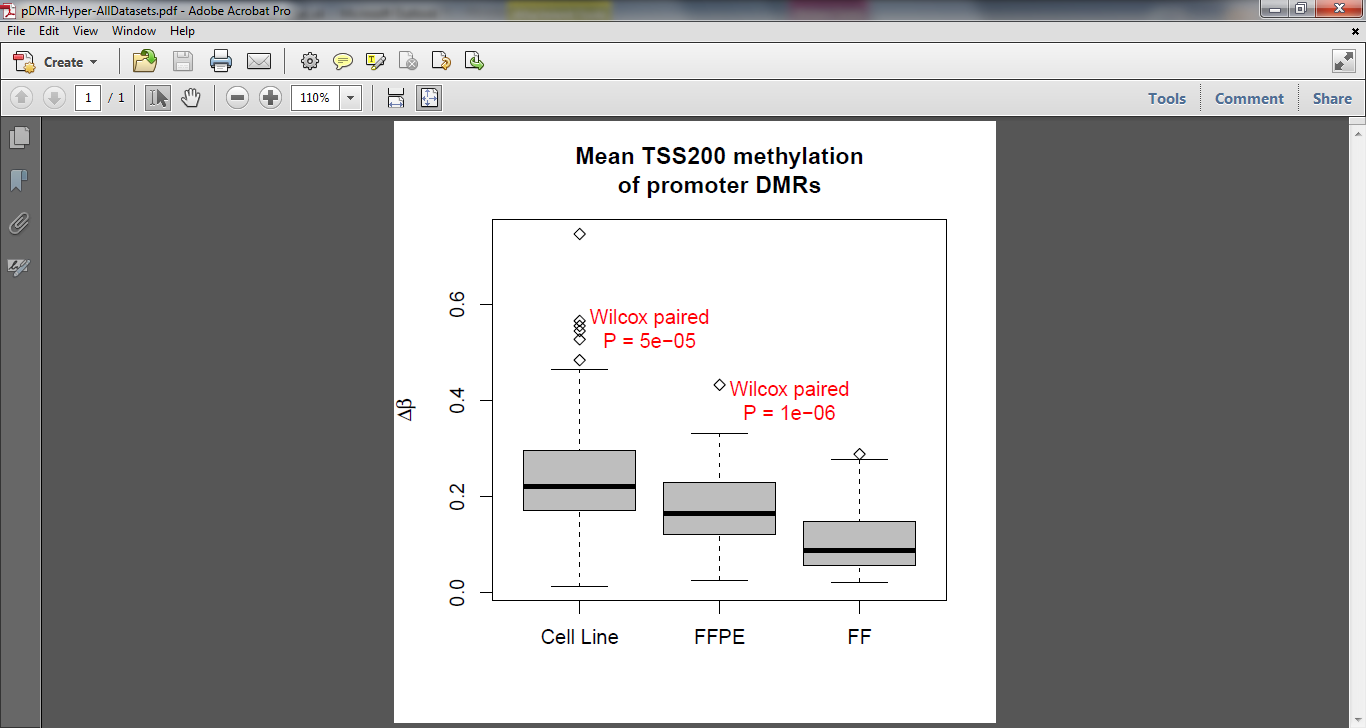


**Additional File 1, Figure S6: Comparison of methylation differences across all datasets.** A) Boxplots of changes in absolute mean beta value (Δβ) of the top 1000 MVPs in HPV+ and HPV– cell lines, FFPE tumours and FF tumours. Methylation changes are substantially larger in cell lines in contrast to the changes detected in FFPE (Paired Wilcox *P* = 7e-15) and again larger than in FF tumour samples (Paired Wilcox *P* = 3e-13). B) Boxplots of changes in mean beta value (Δβ) of promoter DMRs in HPV+ and HPV– cell lines, FFPE tumours and FF tumours. HPV cell lines showed the largest changes in TSS200 methylation, significantly larger than FFPE (Paired Wilcox *P* = 5e-05) which showed significantly higher methylation relative to FF (Paired Wilcox *P* = 1e-06).

**Additional File 1, Figure S7: RT-qPCR analysis of DNA methyltransferase expression in HPV+ cell lines, compared with HPV- cell lines.** Error bars represent standard deviations of the mean fold change (3HPV+ lines vs 3HPV- lines) across three independent experiments. ** p<0.01; ***p<0.001 (T-test, HPV+ lines versus HPV- lines).


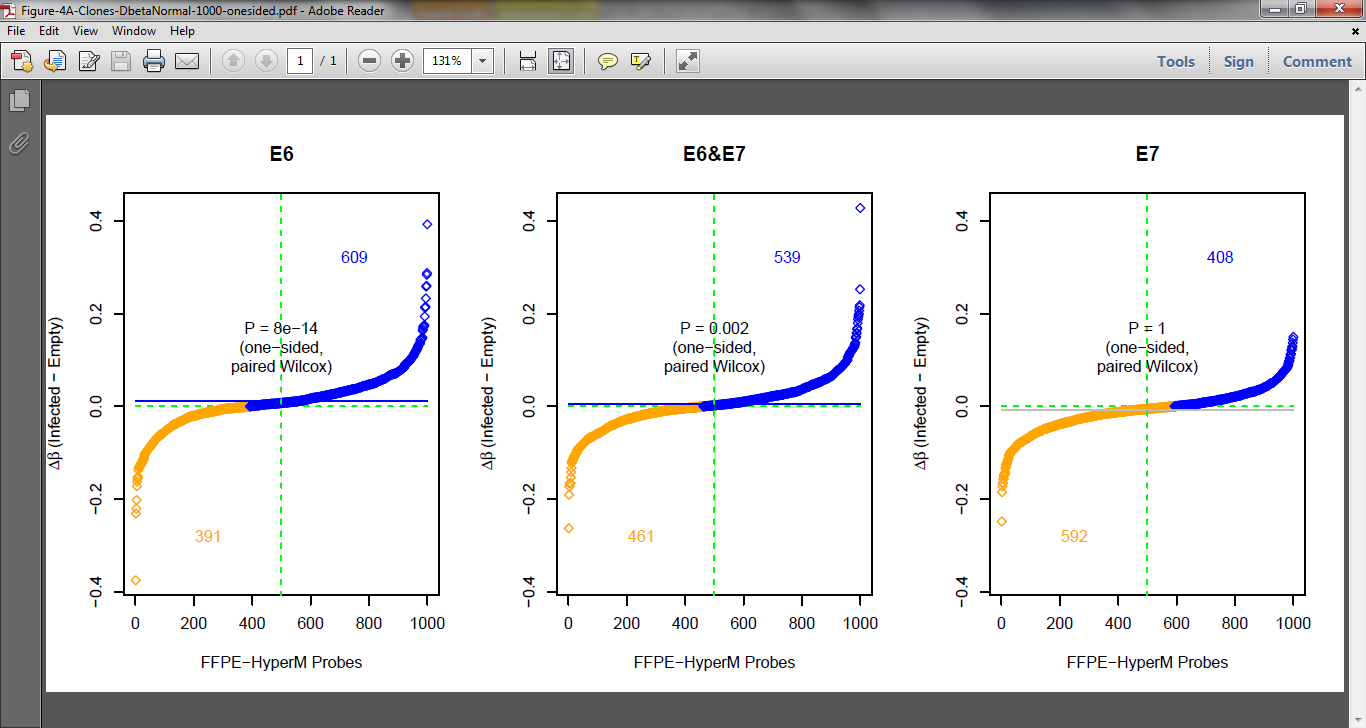


**Additional File 1, Figure S8: Scatterplots of change in mean methylation change Δβ (*y*-axis) against hypermethylated probes in FFPE samples (*x*-axis).** A one-sided Wilcox test of top 1000 FFPE probes (annotating across all datasets) illustrating that the hypermethylation signature observed in FFPE is partially recapitulated by E6 infected and E6&E7 infected clones. The mean of the distribution is marked with a horizontal and the total number of probes in each class is annotated in the appropriate colour (hypomethylated: orange; hypermethylated: blue).


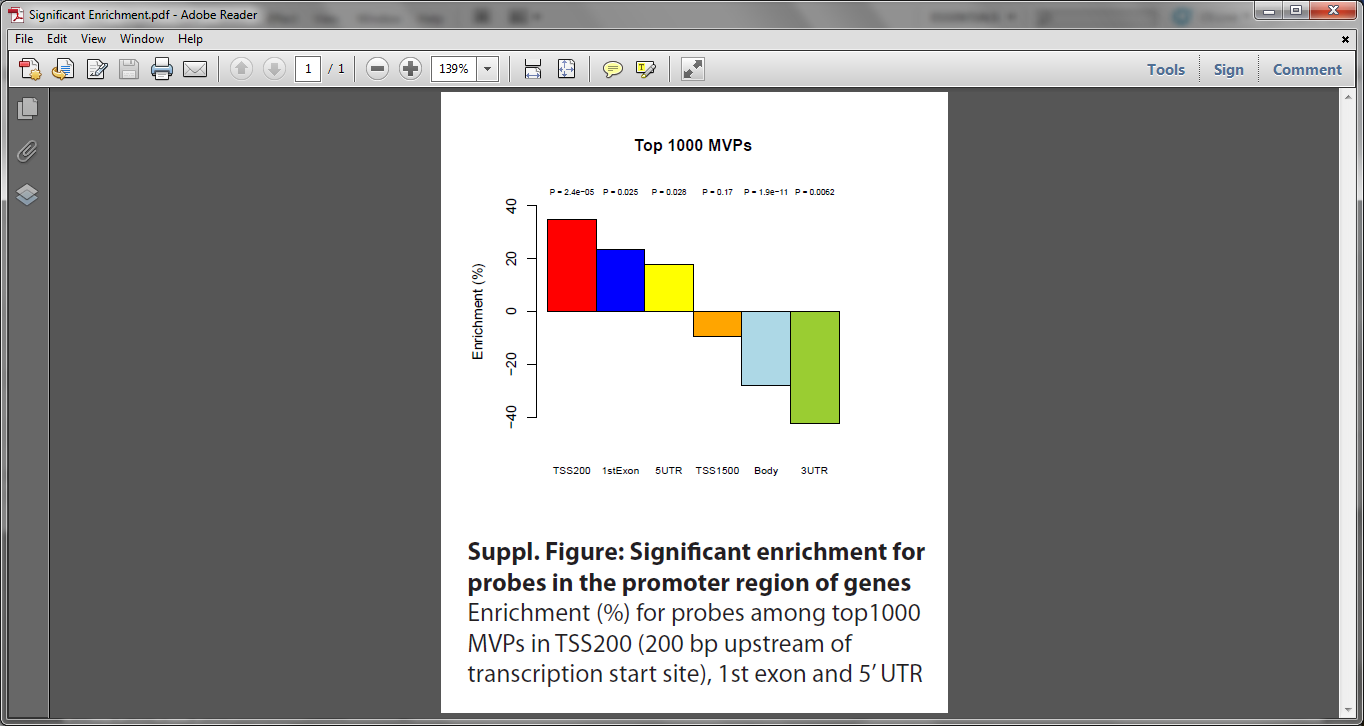


**Additional File 1, Figure S9:** **Enrichment (%) of hypermethylation of promoter regions amongst MVPs.** Significant over-representation of the TSS200 (35%), 1stExon (23%) and 5’UTR (18%) regions amongst the top 1000 MVPs (*P* < 0.05 in each case) and significant under-representation in the Body (–28%) and 3’UTR (–42%)


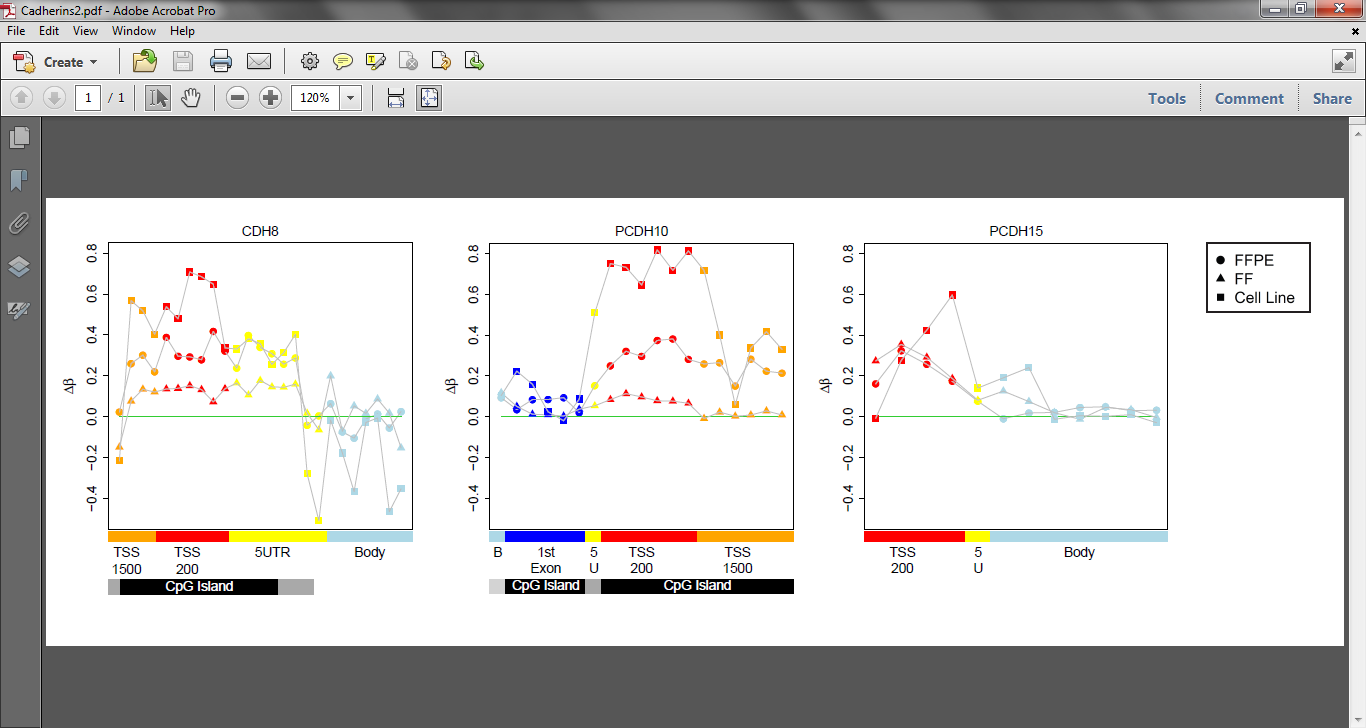


**Additional File 1, Figure S10:** **Profiles of hypermethylated TSS200 promoter DMRs of cadherin genes** (across FFPE HNSCCs, FF HNSCCs and HNSCC cell lines), namely CDH8, PCDH10 and PCDH15. Feature annotation is as provided by BeadChip and methylation values are colour-coded accordingly: TSS1500 = orange (1500 bp – 200 bp upstream of the transcription start site), TSS200 = red (200 bp upstream of the transcription start site), 5’UTR = yellow, gene body = blue, CpG islands = black, CpG shores = grey and CpG shelves = light grey.

**Additional File 1, Figure S11: RT-qPCR analysis of CDH8 and PCDH10 expression in HPV- cell lines compared with HPV+ cell lines.** CDH8 and PCDH10 are significantly overexpressed in a group of three HPV negative HNSCC cell lines relative to three HPV positive cell lines, consistent with hypermethylation in the latter. Error bars represent standard deviations of the mean fold change (3HPV+ lines vs 3HPV- lines) across three independent experiments. *** p<0.001 (T-test: HPV- lines versus HPV+ lines).
