# Supplementary material for: Identification and functional validation of HPV-mediated hypermethylation in head and neck squamous cell carcinoma
Source: Genome Med. 2013 Feb 5;5(2):15. doi: 10.1186/gm419 (PMC3706778; doi:10.1186/gm419)
Supplement: Additional file 2 — Supplemental tables. [file gm419-S2.DOCX]

|  | HPV+ (n=21) | HPV- (n=21) |
| --- | --- | --- |
| Median Age (range) | 56.9 years (42-81) | 58.4 years (43-77) |
| Gender | M: 14  F: 7 | M: 17  F: 4 |
| Tumor site | Oropharynx: 21 | Oropharynx: 21 |
| Tumor grade | Well diff: 1  Mod diff: 10  Poorly diff: 10 | Well diff: 0  Mod diff: 15  Poorly diff: 6 |
| Tumor stage (T) | T1: 5  T2: 7  T3: 4  T4: 3  N/a: 2 | T1: 1  T2: 3  T3: 8  T4: 9  N/a: 0 |
| Cervical lymph node involvement (N) | Yes: 16  No: 2  N/a: 3 | Yes:16  No: 5  N/a: 0 |
| Smoking | Ever: 8  Never: 10  N/a: 3 | Ever: 17  Never: 0  N/a: 4 |
| Alcohol | Heavy drinker: 2  Occ. alcohol: 6  Never: 4  N/a: 9 | Heavy drinker: 13  Occ. alcohol: 3  Never: 1  N/a: 4 |

**Additional File 2, Table S1: Patient characteristics of all 42 FFPE cases.** It is well known that HPV-positive HNSCC patients are frequently younger than HPV-negative HNSCC patients. We therefore took great care to match the samples for age. The mean age in the HPV-positive group was 56.9 years and 58.4 years in the HPV-negative group.

|  | HPV+ (n=3) | HPV- (n=3) |
| --- | --- | --- |
| Age | 58.3 years (53-62) | 73.6 years (66-80) |
| Gender | M: 2  F: 1 | M: 3  F: 0 |
| Tumour site | Oropharynx: 3 | Oropharynx: 3 |
| Tumour grade | Well diff: 0  Mod diff: 0  Poorly diff: 3 | Well diff: 0  Mod diff: 1  Poorly diff: 2 |
| Tumour stage (T) | T1: 0  T2: 3  T3: 0  T4: 0  N/a: 0 | T1: 0  T2: 0  T3: 0  T4: 2  N/a: 1 |
| Cervical lymph node involvement (N) | Yes: 3  No: 0  N/a: 0 | Yes: 0  No: 2  N/a: 1 |
| Smoking | Ever: 3  Never: 0  N/a: 0 | Ever: 2  Never: 0  N/a: 1 |
| Alcohol | Heavy drinker: 1  Occ. alcohol: 2  Never: 0  N/a: 0 | Heavy drinker: 0  Occ. alcohol: 2  Never: 1  N/a: 0 |

**Additional File 2, Table S2: Patient characteristics of fresh frozen samples.**

| **Sample name** | **p16 staining** | **E6 qPCR** |
| --- | --- | --- |
| P3 | neg | neg |
| P6 | pos | pos |
| P7 | neg | neg |
| P8 | pos | pos |
| P9 | neg | neg |
| P10 | neg | neg |
| P11 | neg | neg |
| P12 | neg | neg |
| P13 | pos | pos |
| P14 | neg | neg |
| P16 | pos | pos |
| P17 | neg | neg |
| P19 | pos | pos |
| P24 | neg | neg |
| P25 | neg | neg |
| P26 | pos | pos |
| P28 | pos | pos |
| P29 | neg | neg |
| P30 | neg | neg |
| P35 | pos | pos |
| P36 | neg | neg |
| P37 | pos | pos |
| P38 | pos | pos |
| P40 | neg | neg |
| P41 | neg | neg |
| P42 | neg | neg |
| P43 | pos | pos |
| P47 | pos | pos |
| P48 | pos | pos |
| P50 | pos | pos |
| P60 | pos | pos |
| P62 | neg | neg |
| P67 | pos | pos |
| P72 | pos | pos |
| P74 | pos | pos |
| P79 | pos | pos |
| P82 | pos | pos |
| P83 | pos | pos |
| P90 | neg | neg |
| P91 | neg | neg |
| P92 | neg | neg |
| P94 | neg | neg |
|  |  | pos: E6 copy number/cell > 0.1 * |

**Additional File 2, Table S3: p16 and E6 qPCR results of HPV+ and HPV- FFPE HNSCC samples selected for experiments.**

* According to Zhao et al., Feasibility of quantitative PCR-based saliva rinse screening of HPV for head and neck cancer; Int J Cancer; 2005; 117(4):605-10.

| **Name of primer/probe** | **Primer/probe Sequence (5'-3')** |
| --- | --- |
| HPV type 16 GAPDH forward primer | 5’- GGAGTCAACGGATTTGGTCGTA -3’ |
| HPV type 16 GAPDH forward primer | 5’- GGCAACAATATCCACTTTACCAGAGT -3’ |
| HPV 16 GAPDH TaqMan probe | 5’-(FAM)- CGCCTGGTCACCAGGGCTGC -(TAMRA)-3’ |

**Additional File 2, Table S4: GAPDH primers and probe used for qPCR.**

| **Name of primer/probe** | **Primer/probe Sequence (5'-3')** |
| --- | --- |
| HPV type 16 E6 forward primer | 5’-TCAGGACCCACAGGAGCG-3’ |
| HPV type 16 E6 reverse primer | 5’-CCTCACGTCGCAGTAACTGTTG-3’ |
| HPV 16 E6 TaqMan probe | 5’-(FAM)-CCCAGAAAGTTACCACAGTT  ATGCACAGAGCT-(TAMRA)-3’ |

**Additional File 2, Table S5: E6 primers and probe used for qPCR.**

| **Sample** | **HPV** | **Cycles** | **Total Reads (pre-alignment)** | **Unique fragments (paired reads)** | **Mean Insert Size** | **CpG Enrichment Score** | **CpG coverage** | **%GC** |
| --- | --- | --- | --- | --- | --- | --- | --- | --- |
| HN_29 | HPV- | 36 | 52655678 | 17002647 | 174.43 | 4.06 | 48% | 52 |
| HN_32 | HPV- | 36 | 58152760 | 19332002 | 189.9 | 3.78 | 54% | 52 |
| HN_39 | HPV+ | 36 | 40889864 | 11829705 | 196.16 | 3.45 | 45% | 50 |
| HN_96 | HPV- | 36 | 41538032 | 12474190 | 185.06 | 3.19 | 47% | 49 |
| HN_105 | HPV+ | 36 | 36259072 | 11385250 | 173.75 | 3.68 | 44% | 52 |
| HN_125 | HPV+ | 36 | 47522856 | 15101872 | 178.68 | 3.96 | 47% | 52 |

**Additional File 2, Table S6: Read counts obtained from the methylome analysis on MeDIP-Seq data.**


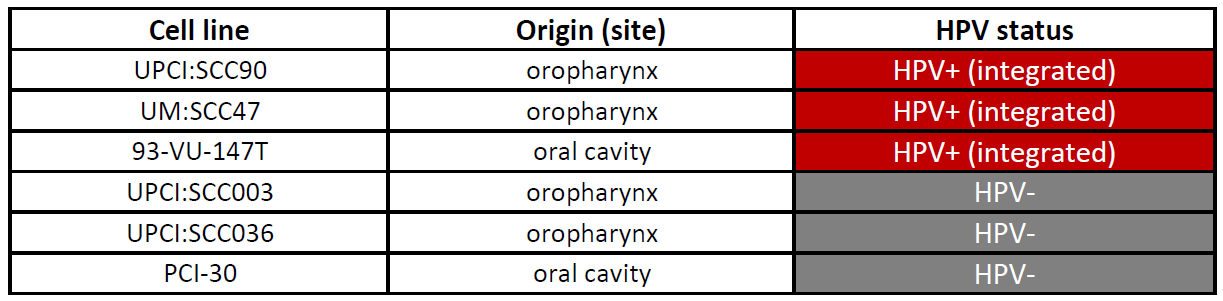


**Additional File 2, Table S7: HPV+ and HPV- HNSCC cell lines used for experiments.**

|  | **Normalized* E6 expression levels** | **Normalized* E7 expression levels** |
| --- | --- | --- |
| **pLXSN-E6 clone 4** | 0.43 | 0.00 |
| **pLXSN-E6 clone 1** | 0.25 | 0.00 |
| **pLXSN-E6 clone 2** | 0.11 | 0.00 |
| **pLXSN-0 clone 2** | 0.00 | 0.00 |
| **pLXSN-0 clone 3** | 0.00 | 0.00 |
| **pLXSN-0 clone 4** | 0.00 | 0.00 |
| **pLXSN-E6/E7 clone 1** | 0.01 | 0.11 |
| **pLXSN-E6/E7 clone 2** | 0.01 | 0.06 |
| **pLXSN-E6/E7 clone 5** | 0.01 | 0.06 |
| **pLXSN-E7 clone 2** | 0.00 | 0.09 |
| **pLXSN-E7 clone 4** | 0.00 | 0.02 |
| **pLXSN-E7 clone 5** | 0.00 | 0.07 |

***Normalized to UPCI:SCC90**

**Additional File 2, Table S8: Normalized expression levels of the viral oncogenes *E6* and *E7* in infected cell line clones** (Expression levels were normalized to those of the HPV+ HNSCC cell line UPCI:SCC90). The lower E6 and E7 expression levels in infected clones, compared with those in UPCI:SCC90, may explain why the hypermethylation signature observed in HPV+ HNSCC tumours and HPV+ HNSCC cell lines was only partially phenocopied in these clones.

| **Gene List** | **nList** | **nRep** | **fRep** | **nOVERLAP** | **ODDS RATIO** | ***P*-value** | **Adj.*P*-value** | **Genes** |
| --- | --- | --- | --- | --- | --- | --- | --- | --- |
| ACEVEDO_METHYLATED_IN_  LIVER_CANCER_DN | **927** | **778** | **0.8392665** | **78** | **2.3886656** | **1.60E-10** | **1.03E-06** | SDK1; TNFRSF8; C20orf151; CNTNAP4; CSRP3; KCNAB2; ITGA8; TREML2; DPP6; KIRREL; OR1C1; PTP4A3; OR6V1; CACNB2; RSU1; RNF17; CA1; TAC1; FAM105A; NFASC; SV2B; FANK1; NAV2; MYO10; CLNK; VPS8; ZAP70; ZNF660; COL24A1; C14orf53; SLC26A4; SORCS3; LIMCH1; CHRM3; DEFB108B; RPTOR; OR2B11; POTEE; TMEM132D; OR10K1; PTPRN2; SPATA18; CASQ2; PXDNL; ST6GALNAC3; MAGEH1; STK32B; RUNX1T1; ESD; A2BP1; JAKMIP1; DOCK3; RGS6; ZNF239; SLCO5A1; IL2RA; GPR139; OLFML2B; CLSTN2; MAL2; GSG1; KIAA1026; DENND3; MYT1L; SPAG11B; ANK2; ADARB2; CNR1; NLRP3; VIPR2; KIF26B; RADIL; FBXW7; ODZ2; SLC6A8; CDH13; ZNF804A; CCDC141; |
| AACTTT_UNKNOWN | **1475** | **1429** | **0.9688136** | **114** | **1.875692** | **1.07E-08** | **3.43E-05** | IGF1; BTBD3; RORA; SSBP3; UST; KIFAP3 ESRRG; SLC4A10; ARID1B; CREB5; SH3BP4; TOM1; NOL4; CACNA1H; HCN1; IQGAP2; GPR65; LSAMP; OTP; NEUROD4; RASSF2; NRXN1; HOXC11; CACNB2; CACNA1D; GPR133; CA3; CA10; TAC1; ROR1; KCNQ4; HCRTR2; NRG1; MSX2; MRVI1; MYBPC1; BCOR; LDB2; SEMA3A; SYNE1; NOX3; LUM; GRIN3A; PCDH10; ZNF219; SNTG1; PRKAG2; TREM1; SORCS3; PDE4D; CHRDL1; KIRREL3; RBMS3; REPS1; PTPRG; SHANK2; TEAD1; CHST8; SFRS8; TECTA; COL19A1; STC1; TRPS1; CAMK2D; CASQ2; RPS6KA2; CNTNAP2; STK32B; HPSE2; CBFA2T3; CTNNA3; ESR1; A2BP1; DOCK3; SESN2; RBPMS; CTNND2; CTNND1; CDYL; LRRTM4; SEMA5A; TRERF1; HOXA2; KCNMA1; DLG2; GFPT2; JAZF1; KCNJ3; SMOC1; NXF1; ELAVL4; CLSTN2; NRK; C10orf11; MRPS31; MAPK10; MAP2K6; NELL1; GABRB1; KCNIP4; MOS; MRGPRF; COL4A6; C5orf4; AKT3; CPNE5; ADCY2; VIPR2; LRRN1; EBF2; FBXW7; SERPINI1; CDH13; CDK5 |
| BENPORATH_SUZ12_  TARGETS  (M9898)  PMID: 18443585 | **1037** | **998** | **0.9623915** | **82** | **1.9049574** | **4.53E-07** | **0.00096895** | PLEC1; ST3GAL1; DPP6; NOL4; KY; CRMP1; OTP; OCA2; PKNOX2; HOXC11; CACNB4; CACNA1D; CA3; CA10; LRRC3B; SIDT1; NRG1; NRCAM; NPTX1; SV2B; EIF4E3; APCDD1L; BATF3; NAV2; NCAM1; GRIN3A; COL24A1; SLC26A4; SORCS3; DKK2; XYLT1; CYFIP1; KIRREL3; PHLDB1; NT5C1A; SLITRK2; LCORL; CTPS2; PRKG1; CHST8; ABCC6; ANKRD11; CSMD1; SPATA18; FGF3; IGSF21; GPC5; RASGRF2; STK32B; HPSE2; ASTN1; MGAT5B; CTNND2; DLC1; KCNMA1; TLX2; USP3; SLCO5A1; KCNJ3; PDE4DIP; EPB41L4A; CFTR; KCNAB1; MT1X CDH23; OLFML2B; CLSTN2; NRK; NELL1; KCNIP4; ZNF365; ST3GAL6; GPR124; COL4A6; DOK6; ADARB2; SLIT1; VIPR2; GPR137B; FAM163A; CDH13; SLC6A3 |
| BENPORATH_ES_WITH_  H3K27ME3 | **1117** | **1077** | **0.9641898** | **86** | **1.8478054** | **7.77E-07** | **0.00124641** | ADAMTS17; LHX3; PLEC1; DTNB; DPP6; NOL4; KY; ENTPD2; CRMP1; OTP; OCA2; PKNOX2; NRXN1; HOXC11; TFAP2D; CACNB4; CACNA1D; CA10; SIDT1; TAC1; NRG1; MSX2; NPTX1; MYB; SV2B; EIF4E3; BATF3; NAV2; NCAM1; MICB; SYNE1; GRIN3A; COL24A1; PRKAG2; SLC26A4; SORCS3; DKK2; XYLT1; CHRDL1; KIRREL3; PHLDB1; NT5C1A; NRIP3; PSD2; PRKG1; CHST8; PTPRN2; SNTB1; CSMD1; MAST4; CAMK2B; FGF3; IGSF21; GPC5; RPS6KA2; PARD3B; STK32B; HPSE2; PDE10A; ASTN1; OSBP2; ESR1; CTNND2; HOXA2; KCNMA1; TLX2; KCNQ1; SLCO5A1; KCNJ3; PDE4DIP; SLC26A5; EPB41L4A; KCNAB1; CDH23; OLFML2B; CLSTN2; MXRA7; NELL1; KCNIP4; GPR124; COL4A6; DOK6; ADARB2; SLIT1 FAM163A; SLC6A3 |
| ACEVEDO_LIVER_CANCER_  WITH_H3K27ME3_UP | **295** | **275** | **0.9322034** | **30** | **2.5446155** | **1.53E-05** | **0.01967642** | C7orf31; SDK1; KIAA1217; COL19A1; MAGEB1; C9; CRISP2; MAST4; OR2H1; TSGA13; MAGEH1; CREB5; CTNNA3; CTNNA3; PRTG; TMEM71; SCAND3; SELP; AKT3; CSRNP3; LIMCH1; TMEM144; RBPMS; CHRM3; OCA2; CACNB2; FMN2; DLC1 ZBTB46; HSPB3; CCDC141 |
| module_100 | **529** | **513** | **0.9697543** | **46** | **2.0597127** | **1.95E-05** | **0.01970417** | BTBD3; DTNB; TRPM2; PLEKHB1; PLEKHB1; CNTNAP2; RUNX1T1; GALR3; DPP6; CRMP1; SORBS2; PTP4A3; C16orf45; SCHIP1; RASSF2; NRXN1; DVL1; SLC23A2; GFPT2; SYT1; ROR1; MAGI2; NPTX1; ABCA3; ELAVL4; SV2B; LDB2; RSBN1; MBP; MAPK10; MYO10; TACC2; NCAM1; SYNE1;  POLR2J; GNG7; FBXL7; SLIT1; ATP2B2; RBMS3; ZBTB20; SERPINI1; LAMA2; FZR1; PTPRN2; CDH18; CDH18; SLC6A3 |
| module_137 | **531** | **515** | **0.9698682** | **46** | **2.0507021** | **2.15E-05** | **0.01970417** | BTBD3; DTNB; TRPM2; PLEKHB1; CNTNAP2; RUNX1T1; GALR3; DPP6; CRMP1; SORBS2 PTP4A3; C16orf45; C16orf45; SCHIP1; RASSF2; NRXN1; DVL1; SLC23A2; GFPT2; SYT1; ROR1; MAGI2; NPTX1; ABCA3; ELAVL4; SV2B; LDB2; RSBN1; MBP; MAPK10; MYO10; TACC2; NCAM1; SYNE1; POLR2J; GNG7; TTLL1; FBXL7; SLIT1; ATP2B2; RBMS3; ZBTB20; SERPINI1; LAMA2; FZR1; PTPRN2; SLC6A3 |
| module_66 | **536** | **520** | **0.9701493** | **46** | **2.0285086** | **2.74E-05** | **0.02070088** | BTBD3; DTNB; TRPM2; PLEKHB1; CNTNAP2; RUNX1T1; GALR3; DPP6; CRMP1; SORBS2; PTP4A3; C16orf45; SCHIP1; RASSF2; NRXN1; DVL1; SLC23A2; GFPT2 SYT1; ROR1; MAGI2; NPTX1; ABCA3; ELAVL4; SV2B; LDB2; RSBN1; MBP; MAPK10; MYO10; TACC2; NCAM1; SYNE1; POLR2J; GNG7 FBXL7; SLIT1; ATP2B2; RBMS3; ZBTB20; SERPINI1; LAMA2; FZR1; PTPRN2; CDH18; SLC6A3 |
| TTANTCA_UNKNOWN | **718** | **680** | **0.9470752** | **56** | **1.882154** | **2.98E-05** | **0.02070088** | ATP10D; IGF1; EMP1; TRPS1; CAMK2D; CASQ2; ESRRG; ATRNL1; CREB5; C7orf33; HPSE2; WHSC1L1; OTP; A2BP1; CNGB3; SESN2; CTNND2; HOXC11; TRERF1; DLC1; HOXA2; NR1H4; USP3; CORO1C; DLG2; JAZF1; TAC1; ROR1; HGF; KRT14; FLT1; ABL2; SLCO1B1; MAL2; GSG1; C10orf11; MYO10; SEMA3A; GABRB1; KCNIP4; COL4A6; MMP3; PDE4D; CPNE5; KIRREL3; RBMS3; BCKDHA; LRRN1; PTPRG; SHANK2; EBF2; FBXW7; SLC9A9; HRASLS; TECTA |
| BENPORATH_EED_TARGETS  (M7617)  PMID: 1844358 | **1062** | **1015** | **0.9557439** | **76** | **1.7076143** | **3.34E-05** | **0.02070088** | PLEC1; NOL4; KY; OTP; OCA2; PKNOX2; HOXC11; TFAP2D; CACNA1D; CA3; CA10; SIDT1; TAC1; NRG1; NRCAM; MAGI2; MSX2; NPTX1; MYB; SV2B; BATF3; NAV2; NCAM1; MICB; GRIN3A; PCDH10; COL24A1; SLC26A4; SORCS3; DKK2; KIRREL3; NT5C1A; NRIP3; SORCS2; LCORL; PSD2; PRKG1; CHST8; COL19A1; CSMD1; CAMK2B; FGF3; IGSF21; GPC5; STK32B; HPSE2 PDE10A; ASTN1; HCCA2; HTR1E; RGS6; CTNND2; BACH2; HOXA2; KCNMA1; TLX2; KCNQ1; SLCO5A1; PDE4DIP; EPB41L4A; CFTR; KCNAB1; MT1X; CDH23; OLFML2B; CLSTN2; NELL1; ZNF365; DENND3; COL4A6; DOK6 ADARB2 SLIT1 VIPR2 FAM163A SLC6A3 |
| module_11 | **526** | **510** | **0.9695817** | **45** | **2.0214817** | **3.55E-05** | **0.02070088** | BTBD3; DTNB; TRPM2; PLEKHB1; CNTNAP2; RUNX1T1; GALR3; DPP6; CRMP1; SORBS2; PTP4A3; C16orf45; SCHIP1; RASSF2; NRXN1; DVL1 SLC23A2; GFPT2; SYT1; MAGI2; NPTX1; ABCA3; ELAVL4; SV2B; LDB2; RSBN1; MBP; MAPK10; MYO10; TACC2; NCAM1; SYNE1; POLR2J; GNG7; LPHN1; FBXL7; ADARB1; SLIT1; ATP2B2; RBMS3; ZBTB20; BCKDHA; SERPINI1; FZR1; PTPRN2 |
| ATGTACA,MIR-493 | **266** | **262** | **0.9849624** | **28** | **2.4825011** | **4.23E-05** | **0.02106791** | BCOR; TRPS1; HIC2; CTDSPL; SV2B; PREX1; RSBN1; ESRRG; NAV2; ARID1B; TMTC1; KCNIP4; CNOT6; SYNE1; DIP2C; SORCS3; DKK2; NRXN3; PDE4D; C16orf45; CTNND2; CTNND1; ADAM10; PSD2; KCNMA1; SPIRE2; DLG2; CDH13 |
| CAGGTG_V$E12_Q6 | **1850** | **1801** | **0.9735135** | **120** | **1.5230387** | **4.27E-05** | **0.02106791** | LY6G5C; ADAMTS17; BTBD3; SSBP3; UST; DTNB; ESRRG; SLC4A10; KCNAB2; TREML2; ENTPD2; CRMP1; CACNA1H; PTP4A3; CNTN5; NRXN3; RASSF2; NRXN1; SYT11; DVL1; CORO1C; SYT1; TAC1; MFAP2; NAV2; TACC2; NCAM1; ITPR2; PRKAG2; XYLT1; NRIP3;  PCDH15; SHANK2; COX6A2; PTPRB; TECTA; COL19A1; STC1; CAMK2B; PGM3; PLEKHB1; VWF; CASQ1; GRIA1; CBFA2T3; PFKFB2; MGAT5B; GCNT3; DOCK3; MICAL2; RGS6; TRERF1; KCNMA1; N4BP1; KCNQ1; DLG2; JAZF1; SMOC1; EPB41L4A; ABL2; ABR; MAPK10; KCNIP4; MRGPRF; ATM; LPHN1; ADAM10; RAPGEF3; SERPINI1; PIGR; WWOX; SDK1; GALR3; NOL4; IQGAP2; YPEL1; BBX; CACNB2; CACNA1D; HIVEP3; CA10; ROR1; KCNQ4; HCRTR2 NRG1; HGF; NRCAM; NPTX1; SV2B; SEMA3A; SYNE1; LUM; PDE1C; KIRREL3; CTPS2; CHRNA7 CHST8; TRPS1; ALDH3B1; RASGRF2; MAPK8IP1; ESR1; KIF13B; FA2H; DHRS9; CTNND1; TLX2; DOCK5; NR1H4; ELAVL4; GALNT2; NRK; GALR1; GABRB1; GPR124; ANK2; EPB41L5; UACA; LRRN1; CDH13 |
| TGTTTGY_V$HNF3_Q6 | **576** | **552** | **0.9583333** | **47** | **1.9443051** | **5.79E-05** | **0.02652603** | CD19; TRPS1; CNTNAP4; GPLD1; ARID1B; CREB5; CNTNAP2; HPSE2; SCG3; CRY2; KIRREL; WHSC1L1; IQGAP2; A2BP1; ESR1; ZDHHC14; BBX; NRXN3; HOXC11; UBASH3A; DLG2; CA3; BANP; HCRTR2; JAM3; SMOC1; NRG1; ELAVL4; MAPK10; SEMA3A; GABRB1; NCAM1; KCNIP4; RBP3; SORCS3; AKT3; PDE3B; ROBO4; PDE4D; ADARB2; CHRDL1; KIRREL3; PTPRG; EBF2; TEAD1; TERT; TNMD |
| RNTCANNRNNYNATTW_UNKNOWN | **48** | **48** | **1** | **10** | **5.40694** | **6.20E-05** | **0.02652603** | LRRN1; CASQ2; ELAVL4; NOL4; NRXN1; CHRDL1; MYB; CDK5; TAC1; USP3 |
| VERHAAK_AML_WITH_NPM1_MUTATED_DN | **246** | **241** | **0.9796748** | **26** | **2.5057869** | **6.70E-05** | **0.02685118** | HGF; FAM105A; EMP1; ROBO1; CD19; CD7; MXRA7 MECOM; RPS6KA2; SH3BP4; STK32B; NCAM1; RUNX1T1; LTF; BAALC; ZAP70; BANK1; YPEL1; PDE3B; XYLT1; GNG7; RBPMS; LHFPL2; THSD7A; NT5E; SIDT1 |
| TTGTTT_V$FOXO4_01 | **1549** | **1489** | **0.9612653** | **101** | **1.542327** | **9.36E-05** | **0.03532313** | BTBD3; RORA; ROBO1; SSBP3; RASGRP3; ESRRG; CRY2; ENTPD1; CRMP1; PKNOX2; RASSF2; CORO1C; NCAM1; PRKAG2; TEAD1; TNMD; STC1; ESM1; GPLD1; VWF; CASQ2; GRIA1; ADORA1; HPSE2; SCG3; CTNNA3; A2BP1; CNGB3; KRTAP9-2; TRERF1; DLC1; KCNJ16; DLG2; SMOC1; KRT14; MAGEB1; FLT1; CLSTN2; MAPK10; MAP2K6; KCNIP4; MRGPRF; SLC12A8; SLC4A4; COL4A6; AKT3; LPHN1; ADARB2; CNR1; TNXB; SCML4; SDK1; IGF1; C20orf151; CNTNAP4; ARID1B; CREB5; KIRREL; OTP; ZDHHC14; YPEL1; SORT1; HCRTR2; NRG1; MSX2; NPTX1; BCOR; LDB2; SEMA3A; MRPL11; NOX3; ZNF219; RBP3; PDE3B; PDE4D; CHRDL1; SLITRK2; PRKG2; CHST8; ATP10D; EMP1; TRPS1; ZNRF2; WHSC1L1; GABARAPL1; SESN2; TLN2; RCL1; CTNND1; LRRTM4; HOXA2; C10orf81; USP3; MGAT4A; CFTR; CDH19; ELAVL4; NRK; TSGA13; ROBO4; EBF2 |
| V$CEBPDELTA_Q6 | **185** | **179** | **0.9675676** | **21** | **2.7470556** | **0.0001005** | **0.03582371** | ANKRD11; OTP; SDK1; A2BP1; ITGA5; RORA; STC1; DOCK3; RAB3IP; VIPR2; GALK2; SYT11; TLX2; SLC4A10; ARID1B; SEMA3A; FBXW7; CREB5; CNTNAP2; C7orf33; TAC1 |
| V$OCT1_02 | **180** | **167** | **0.9277778** | **20** | **2.8105184** | **0.0001097** | **0.03703975** | JAM3; OTP; STC1; NRXN3; TRPS1; PDE4D; ELAVL4; HIC2; PKNOX2; HOXC11; CHRDL1; KIRREL3; NRK ESRRG; DNAH9; CACNA1D; SEMA3A; DLG2; KCNIP4; ENTPD1 |
| V$MEIS1_01 | **175** | **168** | **0.96** | **20** | **2.7913797** | **0.0001192** | **0.03822413** | SMOC1; NRG1; A2BP1; ANK2; NEUROD4; ABL2; UST; RGS6; ELAVL4; COL11A1; KIRREL3; PHLDB1; ESRRG; FBXW7; CREB5; SLC23A2; NCAM1; NOL4; MRGPRF; HCRTR2 |
| ACEVEDO_LIVER_CANCER_WITH_H3K9ME3_UP | **141** | **122** | **0.8652482** | **16** | **3.1109729** | **0.0001824** | **0.05570731** | FAM105A; ANK2; NRXN3; LIMCH1; KRT20; ENOX1 SULT1C2; TRPM2; CACNB2; LRRN1; FAT3; MAGEH1; MYO16; CLNK; TMEM108; CA1 |
| SCHUETZ_BREAST_CANCER_DUCTAL_INVASIVE_UP | **346** | **331** | **0.9566474** | **31** | **2.1434204** | **0.0002001** | **0.05831783** | JAM3; ATP10D; EMP1; ROBO1; ESM1; OLFML2B; CRISPLD2; MFAP2; COL11A1; LDB2; CLEC4A; MXRA7; HEG1; PDE10A; ENTPD1; LUM; GPR65 COL6A3; COL8A2; ST3GAL6; MMP3; ANKRD6; AKT3; MICAL2; XYLT1; HLA-DRB6; RASSF2; FAP; THSD7A; GFPT2; ROR1 |
| TGATTTRY_V$GFI1_01 | **243** | **230** | **0.9465021** | **24** | **2.4098435** | **0.0002096** | **0.05844938** | STC1; MYBPC1; ELAVL4; CLSTN2; LDB2; CASQ2; C10orf11; ESRRG; MAPK10; RASGRF2; CREB5; CTNNA2; LSAMP; ESR1; NRXN3; CTNND1; NRXN1; CHRDL1; TRERF1; HOXA2; TEAD1; JAZF1; HIVEP3; CDH13 |
| SMID_BREAST_CANCER_BASAL_DN | **692** | **667** | **0.9638728** | **52** | **1.7658389** | **0.0002251** | **0.06014118** | IGF1; EFCAB2; MAST4; STS; CAMK2B; ICA1; PCLO; ESRRG; ATRNL1; ZNF385D; CNTNAP2; STK32B CBFA2T3; FAM174B; CRY2; CACNA1H; IQGAP2; ESR1; KIF13B; NME5; C16orf45; MTL5; KCNMA1; CACNA1D; KCNJ3; SYT1; PSCA; SIDT1; DARC; HGF; CYBRD1; MSX2; MYB; MCF2L; KCNAB1; ABCA3; CLSTN2; KDM4B; GGT1; ZNF91; ZSCAN18; GOLSYN; COL4A6; C5orf4; DNALI1; FBXL7; CNR1; CNR1; RAPGEF3; LMF1 SERPINI1; PTPRN2; WWOX |
| DELYS_THYROID_CANCER_DN | **214** | **205** | **0.9579439** | **22** | **2.4841333** | **0.0002552** | **0.06546773** | CYBRD1; KCNAB1; MT1X; LDB2; CASQ2; PCLO; ESRRG; NCAM1; DPP6; LTF; PDE10A; SYNE1; IQGAP2 SLC4A4; C5orf4; SLC26A4 CHRDL1; DLG2; TNXB; LAMA2; WWOX; FXYD6 |
| BENPORATH_PRC2_TARGETS  (M8448)  PMID: 18443585 | **652** | **625** | **0.958589** | **49** | **1.7742791** | **0.0002977** | **0.07344045** | CSMD1; PLEC1; FGF3; IGSF21; GPC5; STK32B; HPSE2; NOL4; KY; ASTN1; OTP; CTNND2; OCA2; PKNOX2; HOXC11; KCNMA1; TLX2; CACNA1D; SLCO5A1; CA10; PDE4DIP; SIDT1; NRG1; EPB41L4A; NPTX1; KCNAB1; CDH23; OLFML2B; CLSTN2; SV2B; BATF3; NAV2; NELL1; NCAM1; GRIN3A; COL24A1; COL4A6; SLC26A4; SORCS3; DKK2; DOK6; ADARB2; SLIT1; KIRREL3; NT5C1A; PRKG1; CHST8; FAM163A; SLC6A3 |
| MORF_CAMK4 | **283** | **265** | **0.9363958** | **26** | **2.2512527** | **0.000311** | **0.07386748** | COL19A1; PGM3; PLEKHB1; SOAT2; GPLD1; ZNF157; SUPT3H; TACC2; NOL4; CDH8; SLC26A4; HTR1E; PDE4D; HOXC11; ATP4B; ATP2B2; PHLDB1; RBMS3; PAPPA2; CDR1; COX6A2; CYP2C19; PTPRB; IPO9; FBXL4; HCRTR2 |
| chr6q14 | **44** | **30** | **0.6818182** | **7** | **6.236973** | **0.0003799** | **0.08701329** | PHIP; NT5E; CNR1; ANKRD6; HTR1E; BACH2; PGM3 |
| MORF_MDM2 | **271** | **255** | **0.9409594** | **25** | **2.2479278** | **0.0004069** | **0.08998674** | COL19A1; FLT1; MFN1; CSRP3; PGM3 PLEKHB1; SOAT2; GPLD1; ZNF157; SUPT3H; MAP2K6 TACC2; PDE10A; HTR1E; HOXC11; ATP4B; ATP2B2; PHLDB1; RBMS3; CDR1; COX6A2; PTPRB; IPO9; FBXL4; HCRTR2 |
| module_117 | **706** | **673** | **0.9532578** | **51** | **1.7097179** | **0.0004991** | **0.10419259** | APOB; TNFRSF8; IGF1; TACR3; MPPED2; VNN1 RASGRP3; CD247; AFM; ICA1; ESRRG; CASP5; GPC5 CREB5; ADORA1; KCNAB2; GYG2; NOL4; GPR65; PHACTR2; CNTN5; KIAA0087; IGJ; ZNF239; SULT1C2; CACNB2; CACNB4; HOXA2; NR1H4; IL2RA; CA1; MAGEB1; SV2B; KIR3DL1; NELL1; LGALS2; LTF P2RY6; ABCB11; CNR1; NLRP3; RAPGEF3; MSH4; VIPR2; ALOX5AP; CHRNA7; CDR1; PRKG1; TERT; APOH; SLC6A3 |

**Additional File 2, Table S9: Gene set enrichment analysis on consistent hyper-MVPs.** O**verlaps** (‘nOVERLAP’) between the detected gene set and gene sets in the Molecular Signatures Database v3.0 (MSigDB; Broad Institute of MIT and Harvard, US) were computed as described previously (Subramanian, Tamayo, et al.; PNAS; 2005; 102; 15545-15550). The top 30 hits are illustrated.

| **Gene List** | **nList** | **nRep** | **fRep** | **nOVERLAP** | **ODDS RATIO** | ***P*-value** | **Adj.*P*-value** | **Genes** |
| --- | --- | --- | --- | --- | --- | --- | --- | --- |
| SLEBOS_HEAD_AND_NECK_CANCER_WITH_HPV_UP (M14132)  PMID: 16467079 | 79 | 77 | 0.9746835 | 6 | 47.736128 | 1.12E-08 | 7.18E-05 | HLTF; STMN1; MEIS1; RPA2; MEI1; MCM2 |
| RORIE_TARGETS_OF_EWSR1_FLI1_FUSION_UP | 25 | 25 | 1 | 3 | 70.874864 | 1.82E-05 | 0.0584143 | POU4F1; HLA-E; EMP3 |
| module_252 | 227 | 222 | 0.9779736 | 5 | 12.567451 | 9.00E-05 | 0.16366696 | STMN1; MEIS1; HLTF; RPA2; POU4F1 |
| GNF2_PTPN6 | 45 | 44 | 0.9777778 | 3 | 38.059007 | 0.0001021 | 0.16366696 | HLA-E; EMP3; LIMD2 |
| KALMA_E2F1_TARGETS | 11 | 11 | 1 | 2 | 112.33382 | 0.0002263 | 0.2902739 | RPA2; MCM2 |
| module_198 | 292 | 287 | 0.982876 | 5 | 9.637248 | 0.000297 | 0.318176 | STMN1; MEIS1; HLTF; RPA2; POU4F1 |
| TMTCGCGANR_UNKNOWN | 102 | 96 | 0.9411765 | 3 | 16.75571 | 0.0010224 | 0.718288 | CCNJ; ADNP; POU4F1 |
| DNA_BINDING | 588 | 576 | 0.9795918 | 6 | 5.8035072 | 0.0010974 | 0.718288 | HLTF; KLF11; MCM2; RPA2; POU4F1; TERT |
| HOMEODOMAIN | 293 | 227 | 0.774744 | 4 | 9.5102122 | 0.0012088 | 0.718288 | ADNP; MEIS1; NKX2-4; POU4F1 |
| LOPES_METHYLATED_IN_COLON_CANCER_DN | 26 | 25 | 0.9615385 | 2 | 44.047005 | 0.0012121 | 0.718288 | RPA2; TERT |

**Additional File 2, Table S10: Gene set enrichment analysis on consistent hypo-MVPs.** O**verlaps** (‘nOVERLAP’) between the detected gene set and gene sets in the Molecular Signatures Database v3.0 (MSigDB; Broad Institute of MIT and Harvard, US) were computed as described previously (Subramanian, Tamayo, et al.;PNAS; 2005; 102; 15545-15550). The top 10 hits are illustrated.
